# Model-free feature screening for categorical outcomes: Nonlinear effect detection and false discovery rate control

**DOI:** 10.1371/journal.pone.0217463

**Published:** 2019-05-31

**Authors:** Qingyang Zhang, Yuchun Du

**Affiliations:** 1 Department of Mathematical Sciences, University of Arkansas, Fayetteville, AR, United States of America; 2 Department of Biological Sciences, University of Arkansas, Fayetteville, AR, United States of America; Universita degli Studi del Piemonte Orientale Amedeo Avogadro, ITALY

## Abstract

Feature screening has become a real prerequisite for the analysis of high-dimensional genomic data, as it is effective in reducing dimensionality and removing redundant features. However, existing methods for feature screening have been mostly relying on the assumptions of linear effects and independence (or weak dependence) between features, which might be inappropriate in real practice. In this paper, we consider the problem of selecting continuous features for a categorical outcome from high-dimensional data. We propose a powerful statistical procedure that consists of two steps, a nonparametric significance test based on edge count and a multiple testing procedure with dependence adjustment for false discovery rate control. The new method presents two novelties. First, the edge-count test directly targets distributional difference between groups, therefore it is sensitive to nonlinear effects. Second, we relax the independence assumption and adapt Efron’s procedure to adjust for the dependence between features. The performance of the proposed procedure, in terms of statistical power and false discovery rate, is illustrated by simulated data. We apply the new method to three genomic datasets to identify genes associated with colon, cervical and prostate cancers.

## Introduction

Feature screening, as a key and inevitable step in many bioinformatics applications, is effective in reducing dimensionality and removing redundant features. Because the quality of selected features may greatly affect the subsequent analysis and conclusions, a reliable screening procedure is essential in practice. In general, the ideal feature screening should have high sensitivity and specificity simultaneously, as too many false positives could result in poor model interpretability while too many false negatives may cause lack of fit and inaccurate prediction. In statistics and bioinformatics literature, there has been a wealth of feature screening techniques that can be roughly classified into two categories, namely model-based screening and model-free screening. The model-based methods often rely on a class of specific models such as generalized linear model and nonparametric regression model [1–4]. However with a large number of predictors, it can be very challenging to specify the model structure without prior information. The model-free methods do not require any parametric assumption or model structure, therefore they are more flexible and more efficient than model-based methods for high-dimensional data [5–7].

Different types of data require different feature screening techniques. For instance, the dependence between a continuous response and continuous features could be quantified by correlation-based measures such as Pearson’s correlation, rank-based correlation, and distance correlation [8–10]. There have been a number of model-free procedures recently developed based on these measures. For instance, Li et al. (2012) developed a rank-based feature selector that is robust to outliers and influential points [5]. Li, Zhong and Zhu (2012) introduced a sure independence screening procedure based on distance correlation [6]. Another type of problem is selecting continuous features for a categorical outcome, which is more common in genomic research. For example, it is often of interest to identify genes associated with cancer or certain cancer subtype. Existing approaches for such data type mainly rely on normal-based tests such as two-sample t test (for binary response), Hotelling’s t test and F test (for multi-category response) [11, 12]. These tests are powerful in detecting the mean difference between phenotypes, however, they have several major drawbacks in real genomic applications. Firstly, these tests are normal-based and only targeting linear effects, thus may fail to detect important nonlinear effects. Nonlinear relations are very common in gene regulatory network [13], therefore should be taken into account for feature screening. Secondly, existing approaches have been mostly relying on some classic multiple testing procedures to control the false discovery rate (FDR), such as Benjamini-Hochberg (BH) procedure [14]. However, such procedures control FDR only when the test statistics are independent or weakly dependent, which might not be the case in gene selection problem (genes are often strongly associated with each other). In this paper, we aimed to develop a model-free screening procedure to overcome the two challenges, namely the nonlinear effect detection and FDR control under feature dependencies. To capture nonlinear associations between a categorical response and continuous features, we transformed the problem to testing the equality of two or multiple distributions, and a recently developed nonparametric test was used to evaluate the statistical significance. In addition, we adapted Efron’s multiple testing procedure to control false discovery rate with feature dependence adjustment.

The remainder of the paper is structured as follows: In Section Methods, we formulate the problem and introduce the two-step procedure including edge-count test and Efron’s multiple testing procedure. In Section Results, we conduct a simulation study to evaluate the performance of the proposed procedure in terms of statistical power and false discovery rate control under various settings. The new method is applied to three real genomic datasets to search genes that differentiate cancer and normal subjects. We discuss the new method with some future work perspectives in Section Discussion and conclude the paper in Section Conclusions.

## Methods

### Problem formulation and edge-count test

We consider a general setting where the outcome variable is discrete with *J* categories (*J* < ∞) and the features are continuous. For example in genomics, the outcome response can be normal/diseased, cancer subtypes or tumor stages and each feature can be the expression level of a gene. Existing model-free screening based on correlation measures [5, 6] were developed for continuous outcomes, therefore not suitable for this problem [15]. In this paper, we introduced a novel graph-based method to select continuous features that are associated with a categorical response. Our method is model-free and does not depend on any hypothesis on the form of dependence. To begin with, let {*X*_1_, …, *X*_*p*_} be *p* features (*p* can be large), and {1, …, *J*} be the sampling space of response variable *Y*. With *N* independent observations of {*Y*, *X*_*i*,1≤*i*≤*p*_}, we test the independence between *Y* and *X*_*i*_, which is equivalent to testing equality of *J* conditional distributions, i.e.,

$$\begin{array}{cc} H_{0i} & :F_{X_{i}|Y=1}\left(x\right)=\ldots =F_{X_{i}|Y=J}\left(x\right),\text{for}\;\text{any}\;x\in R\\ H_{\alpha i} & :F_{X_{i}|Y=j}\left(x\right)\neq F_{X_{i}|Y=j^{{}^{\prime }}}\left(x\right),\text{for}\;\text{some}\;x\;\text{and}\;\left(j,j^{{}^{\prime }}\right), \end{array}$$

where $F_{X_{i}|Y=j}(x)$ stands for the cumulative distribution function of *X*_*i*_ in group *Y* = *j*. To test if *H*_0*i*_ is true, we employed a modified edge-count test which is proved more powerful in detecting difference between multiple multivariate distributions [13, 16, 17]. This test has resulted in several successful applications. For instance, Zhang (2018) [13] applied this method to search differentially co-expressed gene pairs from high-dimensional data. Zhang, Mahdi & Chen (2017) [17] employed this test to identify pathways that contribute to ovarian cancer progression. The motivation of the edge-count test is that if samples in difference groups have different distributions, they would be preferentially closer to others from the same group than those from the other group. The distance between samples can be represented by a regular similarity graph. For instance, Chen and Friedman (2017) [16] suggested a minimum spanning tree (MST) or a more general d-MST (a union of d disjoint MSTs). The edge-count test rejects the null hypothesis if the number of between-group edges in the similarity graph is significantly less than what we expected. To implement the graph-based test, we first pooled samples from all *J* groups and indexed them by $1,2,\ldots ,N=\sum_{j=1}^{J}n_{j}$. The group index for sample *k* was denoted by *y*_*k*_. A d-MST is then constructed on the pooled samples using the standard Kruskal’s algorithm [18]. Unless otherwise specified, *G* simultaneously represents the similarity graph and the set of all edges, while |*G*| denotes the total number of edges throughout the paper. For the edge connecting samples *k* and *k*′, i.e., (*k*, *k*′), we define *R*_*j*_ as the number of edges connecting samples from same group *j*, i.e.,

$$\begin{array}{c} R_{j}=\sum_{(k,k^{{}^{\prime }})\in G}I\left(y_{k}=y_{k^{{}^{\prime }}}=j\right), \end{array}$$ (1)

and the test statistic has the following quadratic form:

$$\begin{array}{c} S≔{\left[R-E\left(R\right)\right]}^{T}V^{-1}\left(R\right)\left[R-E\left(R\right)\right], \end{array}$$ (2)

where ***R*** = (*R*_1_, …, *R*_*J*_)^*T*^, ***V***^−1^(***R***) represents the inverse covariance matrix of ***R***. The test statistic defined here simply quantifies the deviation of (*R*_1_, …, *R*_*J*_) from their expected values under permutation null, i.e., $H_{0}^{*}$. Chen and Friedman (2017) [16] established the asymptotic distribution of *S* for *J* = 2, $S\to \chi_{df=2}^{2}$. In our technical report [17], it was proved that the test statistics for *J* groups asymptotically follows a Chi-square distribution with *J* degrees of freedom under mild regularity conditions. To illustrate the results, for an edge *e* in graph *G*, we let

$$\begin{array}{cc} A_{e} & =\left\{e\right\}\cup \left\{e^{\prime }\in G:e^{\prime }\;\text{and}\;e\;\text{share}\;\text{a}\;\text{node}\right\},\\ B_{e} & =A_{e}\cup \left\{e^{\prime \prime }\in G:\exists \;e^{\prime }\in A_{e},\;\text{such}\;\text{that}\;e^{\prime \prime }\;\text{and}\;e\;\text{share}\;\text{a}\;\text{node}\right\}, \end{array}$$

then the following theorem can be derived:

**Theorem 1**. *If* |*G*| = *O*(*N*), $\sum_{k=1}^{N}{\left|G_{k}\right|}^{2}-\frac{{4|G|}^{2}}{N}=O\left(N\right)$, ∑_*e*∈*G*_|*A*_*e*_||*B*_*e*_| = *o*(*N*^3/2^), $\lim_{N\to \infty }\frac{N_{j}}{N}=\lambda_{j}\in \left(0,1\right)$, *then*

$$\begin{array}{c} S≔{\left[R-E\left(R\right)\right]}^{T}V^{-1}\left(R\right)\left[R-E\left(R\right)\right]\overset{D}{\to }\chi_{J}^{2}, \end{array}$$

*where j* = 1, …, *J is the group index*.

The expected values and covariance matrix of (*R*_1_, …, *R*_*J*_) can be derived as follows:

$$\begin{array}{cc} E{\left(R_{j}\right)}_{1\leq j\leq J} & =\left|G\right|\frac{n_{j}\left(n_{j}-1\right)}{N(N-1)},\\ V{\left(R_{j}\right)}_{1\leq j\leq J} & =E\left(R_{j}\right)\left(1-E\left(R_{j}\right)\right)+2C\frac{n_{j}\left(n_{j}-1\right)\left(n_{j}-2\right)}{N(N-1)(N-2)}\\ & +(|G|(|G|-1)-2C)\frac{n_{j}\left(n_{j}-1\right)\left(n_{j}-2\right)\left(n_{j}-3\right)}{N(N-1)(N-2)(N-3)},\\ Cov{\left(R_{j},R_{j^{{}^{\prime }}}\right)}_{j\neq j^{{}^{\prime }}} & =\left(|G|(|G|-1)-2C\right)\frac{n_{j}n_{j^{{}^{\prime }}}\left(n_{j}-1\right)\left(n_{j^{{}^{\prime }}}-1\right)}{N(N-1)(N-2)(N-3)}-E\left(R_{j}\right)E\left(R_{j^{{}^{\prime }}}\right), \end{array}$$

where $N=\sum_{j=1}^{J}n_{j}$ and $C=\frac{1}{2}\sum_{k=1}^{N}|G_{k}{|}^{2}-\left|G\right|$.

The convergence rate of the asymptotic result is the usual *n*^−1/2^ and there are three conditions on the similarity graph (stated in the main Theorem above). |*G*| ∼ *O*(*N*) requires that the density of the graph is of the same order as the pooled sample size. $\sum_{k=1}^{N}{\left|G_{k}\right|}^{2}∼O\left(N\right)$ ensures that there is no large hubs nor many small hubs. ∑_*e*∈*G*_|*A*_*e*_||*B*_*e*_| ∼ *o*(*N*^3/2^) requires there is no cluster of small hubs [16]. These conditions are satisfied by the k-MST based on Euclidean distance [16], we therefore recommend using k-MST as the similarity graph in edge-count test. Furthermore, we conducted a simulation study to evaluate the finite sample performance of the asymptotic null distribution under different sample sizes and different similarity graphs. Details of the simulation settings can be found in S1 File, and the results were summarized in Fig T in S1 File. It is found that under two different models (standard normal distribution and exponential distribution with λ = 1), the asymptotic chi-squared distribution works quite well in approximating p-values, even for relatively small sample size, e.g, 20 samples in each group of *Y*. Increasing sample size generally results in better accuracy of approximation, and the use of slightly denser graph (e.g., 3-MST or 5-MST) may result in better accuracy. These findings are consistent with the simulation results for two groups (*J* = 2) [16]. For small sample sizes (e.g., *n*_*j*_ ≤ 10, *j* = 1, …, *J*), however, the asymptotic distribution might not work well, and in such cases, it is safer to use a permutation p-value based on our test statistic *S*.

It is noteworthy to mention that the main theorem also applies to multi-dimensional features (*X*_*i*_ can be a random vector), i.e., our method can be used to select feature sets. One interesting application is to search biological pathways or gene sets that are associated with certain phenotypes [17]. In addition to the aforementioned edge-count test, some other tests for equality of distributions may also be considered, including Kolmogorov-Smirnov (KS) test [19] and traditional graph-based test [20, 21]. However, these methods have practical limitations in real applications. For instance, KS test is known to be very conservative, i.e., the null hypothesis is too often not rejected [22, 23] (see our simulation study in S1 File for illustrating the conservativeness of KS test). Moreover when the feature is multi-dimensional, the implementation of KS test can be prohibitively computationally intensive. Graph-based tests such as the traditional edge-count tests are easy to implement but they could be problematic under certain location and scale alternatives. As reported recently [16], the traditional edge-count test works well for location alternative under low dimension, however, it becomes problematic for scale alternative (or location+scale alternative, i.e., the two distributions are different in both location and scale), especially when the dimension is moderate to high. This is caused by the fact that the number of within-sample edges in the inner layer would be larger than its null expectation, while the number of within-sample edges in the outer layer would be less than its null expectation, making the edge-count test have low or even no power [16].

### Multiple testing with dependence-adjustment

As we discussed in the previous sections, the prevailing Benjamini-Hochberg procedure may fail to control the false discovery rate in the presence of moderate or strong feature dependence. In the feature screening problem, the test statistics {*S*_1_, …, *S*_*p*_} are correlated under feature dependencies, therefore the BH procedure is not appropriate. To overcome the issue, we adapted a dependence-adjusted multiple testing procedure suggested by Efron (2007) [24]. Unlike the BH procedure, Efron’s procedure does not rely on the independence assumption and generally applies to any dependency structure. It has been extensively studied and widely applied by the statistic community. For instance, Liu (2013, 2017) employed this procedure as a key step to control false discovery rate in the Gaussian graphical model estimation and differential network estimation [25, 26]. To implement Efron’s method, we first transformed the test statistics {*S*_1_, …, *S*_*p*_} into z-values by quantile normalization

$$\begin{array}{c} z_{i}=\Phi^{-1}\left(\text{P}\left(\chi_{df=J}^{2}\leq S_{i}\right)\right),\;\;i=1,...,p, \end{array}$$

where Φ^−1^(⋅) represents the inverse cumulative distribution function of *N*(0, 1). Following the notations in Efron (2007), let $A=\left(P_{0}-{\hat{P}}_{0}\right)/Q_{0}$, where *P*_0_ = 2Φ(1) − 1, ${\hat{P}}_{0}=\sum_{i=1}^{p}I\{|z_{i}\left|\leq 1\right\}/p$, $Q_{0}=1/\sqrt{\pi e}$. In addition, we let

$$\begin{array}{c} A\left(z\right)=\{1+\left|A\right|\frac{\left|z|\phi (z\right)}{\sqrt{2}\left(1-\Phi \left(z\right)\right)}{\}}^{-1}, \end{array}$$

where *ϕ*(⋅) represents the probability density function of *N*(0, 1). Here, *A*(*z*) is used to control the influence of correlation between test statistics (under independence and sparsity, *A*(*z*) is close to 1, thus the procedure is same as BH procedure). The critical value can be obtained as follows:

$$\begin{array}{c} z_{0}=\inf\{-\infty <z<\infty ,1-\Phi \left(z\right)\leq \frac{\alpha A(z)}{p}\max\left(1,\sum_{i=1}^{p}I\left\{z_{i}\geq z\right\}\right)\}. \end{array}$$

To control the FDR at the level of *α* (e.g., *α* = 0.05 or *α* = 0.10), one can solve for the cutoff *z*_0_ and reject $H_{i0}^{*}$ if *z*_*i*_ > *z*_0_. This testing procedure asymptotically controls the FDR at the desired level under some mild regularity conditions (though it might be slightly conservative for some cases) and it works well under all settings in our simulation study. The detailed proof and regularity conditions for Gaussian case can be found in Liu (2017) ([26], see Theorems 3.1 and 3.3).

## Results

### Simulation studies

**Fig 1:**
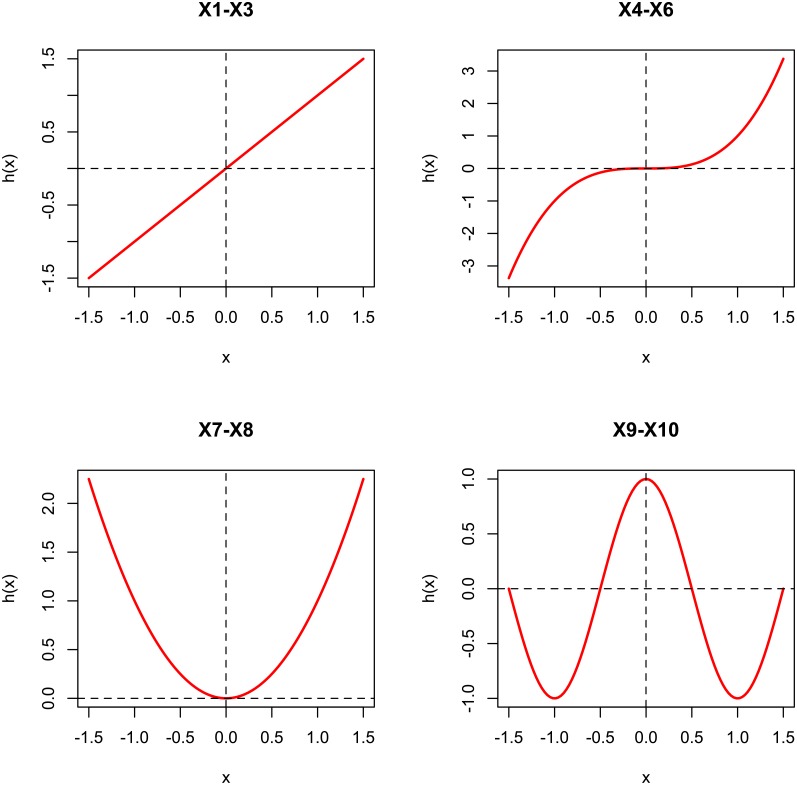
Four transformation functions in the simulation study.

The simulation studies in this part examined the performance of the proposed procedure under several different settings. Without loss of generality, we considered a binary outcome variable *Y* ∈ {0, 1} (i.e., *J* = 2) and *p* continuous features {*X*_1_, …, *X*_*p*_} with sample size *N* (*p* ≥ *N*). Four high-dimensional settings (each setting refers to a combination of model and feature dependency structure) were used to generate the data. To be precise, let *k* ∈ {1, 2, …, *N*} be the index of sample, and *i* ∈ {1, 2, …, *p*} be the index of feature, where we set *p* = 500 and *N* = 50, 100, 200, 500 respectively. In addition, we assumed that only the first 10 features, {*X*_1_, …, *X*_10_}, were associated with *Y* and the other 490 features were redundant. The transformation functions {*h*_*i*_(*X*_*ik*_), 1 ≤ *i* ≤ 10} were set as *h*_*i*_(*X*_*ik*_) = *X*_*ik*_ for 1 ≤ *i* ≤ 3 (linear transformation), $h_{i}\left(X_{ik}\right)=X_{ik}^{3}$ for 4 ≤ *i* ≤ 6 (nonlinear monotonic transformation), $h_{i}\left(X_{ik}\right)=X_{ik}^{2}$ for 7 ≤ *i* ≤ 8 (nonlinear non-monotonic transformation) and *h*_*i*_(*X*_*ik*_) = sin(2*πX*_*ik*_/3) for 9 ≤ *i* ≤ 10 (nonlinear non-monotonic transformation), representing a combination of linear effects and nonlinear effects. The four transformation curves were shown in Fig 1.

To establish the relation between *Y* and {*X*_1_, …, *X*_10_}, we considered two different models:

- Logistic regression model: *Y*_*k*_ ∼ *Bernoulli*(*π*_*k*_), $\log\left\{\pi_{k}/\left(1-\pi_{k}\right)\right\}=\sum_{i=1}^{10}\beta_{i}h_{i}\left(X_{ik}\right)$, *β*_1_ = *β*_2_ = *β*_4_ = *β*_6_ = *β*_7_ = *β*_9_ = 0.5, and *β*_3_ = *β*_5_ = *β*_8_ = *β*_10_ = −0.5
- Latent variable model: $Y_{k}=I\left\{Y_{k}^{*}>0\right\}$, where $Y_{k}^{*}=\sum_{i=1}^{10}\beta_{i}h_{i}\left(X_{ik}\right)+\epsilon_{k}$, *ϵ*_*k*_ ∼ *N*(0, 0.5^2^), *β*_1_ = *β*_2_ = *β*_4_ = *β*_6_ = *β*_7_ = *β*_9_ = 0.5, and *β*_3_ = *β*_5_ = *β*_8_ = *β*_10_ = −0.5

Furthermore, to evaluate the effect of feature dependencies on statistical power and FDR control, we generated the data by two methods:

- Independent features: *X*_*ik*_ ∼ Unif(−1.5, 1.5) for 1 ≤ *i* ≤ 500.
- Dependent features: $X_{ik}=\sqrt{2}Z_{ik}$, where {*Z*_*ik*_}_1≤*i*≤500_ ∼ ***N***_500_(**0**, **Σ**) and **Σ** is a random correlation matrix containing both positive and negative elements (generated by R package *clusterGeneration*). In addition, we conducted an interval truncation (between -1.5 and 1.5) for the samples to avoid extreme values.

The following six testing procedures were applied to each combination of model and feature dependency structure above, namely logistics model with independent features, logistic model with dependent features, latent variable model with independent features and latent variable model with dependent features:

- Edge-count test with Efron’s multiple testing procedure
- Edge-count test with Benjamini-Hochberg procedure
- Welch’s t test with Efron’s multiple testing procedure
- Welch’s t test with Benjamini-Hochberg procedure
- Mutual information z-test with Efron’s multiple testing procedure
- Mutual information z-test with Benjamini-Hochberg procedure

In the edge-count test, a 3-MST was constructed as the similarity graph for better approximation of p-values [17]. To implement Welch’s t test with dependence-adjusted multiple testing, we first calculated and transformed the test statistics into z values via quantile normalization:

$$\begin{array}{c} z_{i}=\Phi^{-1}\left(\text{P}\left(t_{df=v_{i}}\leq t_{i}\right)\right),\;\;i=1,\ldots ,p, \end{array}$$

where the degree of freedom *v*_*i*_ was approximated by Welch-Satterthwaite equation and the test statistics *t*_*i*_ was calculated by the standard formula for t test with unequal variances:

$$\begin{array}{c} v_{i}=\frac{{\left(\frac{s_{i1}^{2}}{n_{1}}+\frac{s_{i0}^{2}}{n_{0}}\right)}^{2}}{\frac{s_{i1}^{4}}{n_{1}^{2}\left(n_{1}-1\right)}+\frac{s_{i0}^{4}}{n_{0}^{2}\left(n_{0}-1\right)}},\;\;t_{i}=\frac{{\bar{X}}_{i1}-{\bar{X}}_{i0}}{\sqrt{\frac{s_{i1}^{2}}{n_{1}}+\frac{s_{i0}^{2}}{n_{0}}}}, \end{array}$$

where {*n*_1_, *n*_0_} stand for the sample sizes for *Y* = 1 and *Y* = 0, $\left\{{\bar{X}}_{i1},{\bar{X}}_{i0}\right\}$ and $\left\{s_{i1}^{2},s_{i0}^{2}\right\}$ represent the sample means and sample standard deviations of *X*_*i*_ in two groups, respectively.

To test whether the mutual information is zero, we used the following Fisher-z transformation:

$$\begin{array}{c} z_{i}^{MI}=\frac{1}{2}\log\frac{1+{\hat{I}}^{*}\left(Y,X_{i}\right)}{1-{\hat{I}}^{*}\left(Y,X_{i}\right)}, \end{array}$$ (3)

where ${\hat{I}}^{*}\left(Y,X_{i}\right)$ represents the normalized sample mutual information between response *Y* and *X*_*i*_, and it can be computed as ${\hat{I}}^{*}\left(Y,X_{i}\right)=\hat{I}\left(Y,X_{i}\right)/\left(\hat{H}\left(Y\right)+\hat{H}\left(X_{i}\right)\right)$, where $\hat{I}\left(Y,X_{i}\right)$ stands for the sample mutual information between *Y* and *X*_*i*_, and $\left\{\hat{H}\left(Y\right),\hat{H}\left(X_{i}\right)\right\}$ stand for the sample entropies of *Y* and *X*_*i*_. By the classical decision theory, $z_{i}^{MI}∼N\left(0,1/\sqrt{N-3}\right)$ under the null hypothesis [27, 28]. The sample mutual information and sample entropy were obtained by R package *infotheo* (https://cran.r-project.org/web/packages/infotheo), where the continuous *X*_*i*_ was discretized into *N*^1/3^ bins.

**Fig 2:**
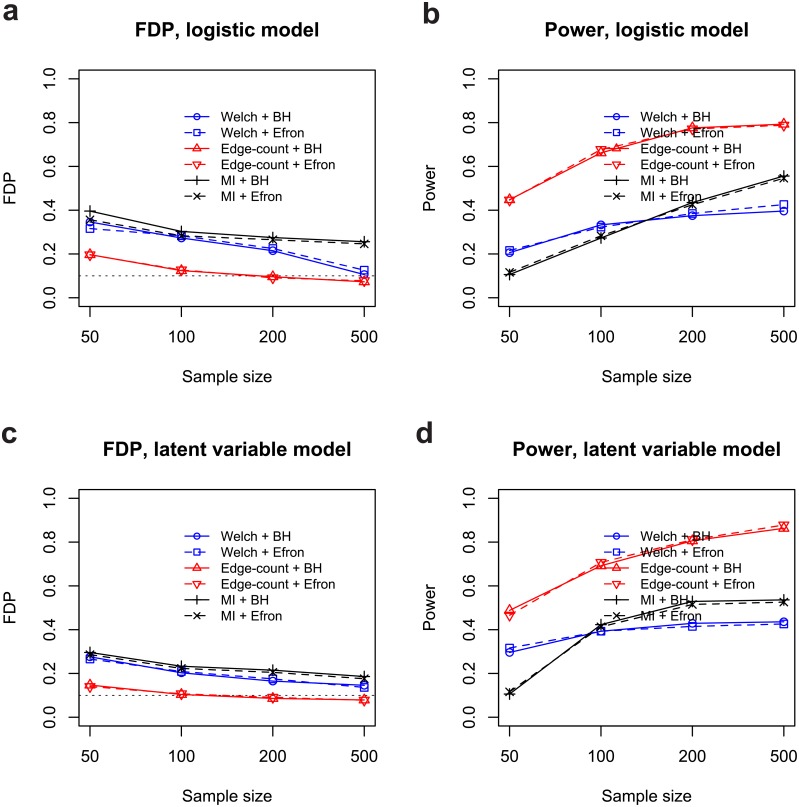
False discovery proportions and empirical statistical powers by six different procedures under independent features: (a) false discovery proportion for logistic model; (b) statistical power for logistic model; (c) false discovery proportion for latent variable model; (d) statistical power for latent variable model. All results were based on 100 replications.

**Fig 3:**
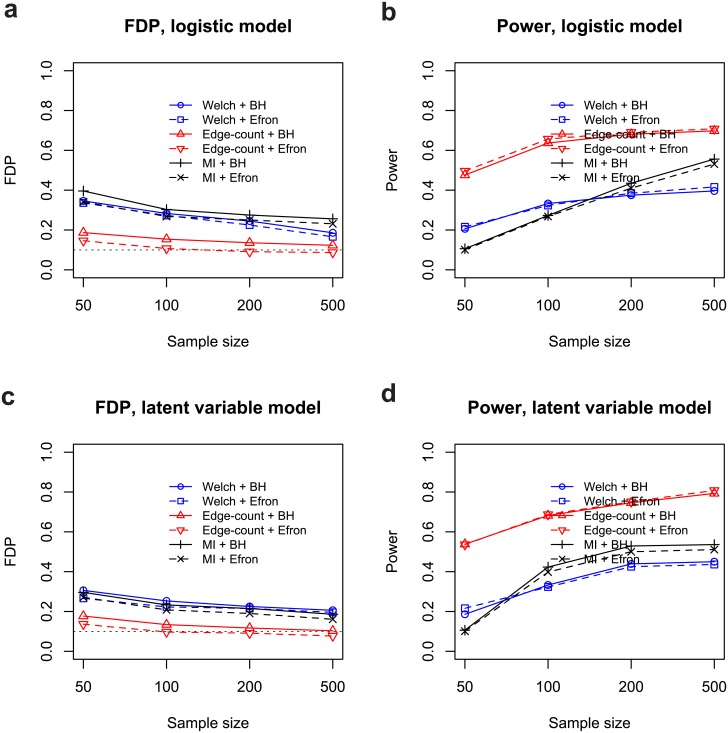
False discovery proportions and empirical statistical powers by six different procedures under dependent features: (a) false discovery proportion for logistic model; (b) statistical power for logistic model; (c) false discovery proportion for latent variable model; (d) statistical power for latent variable model. All results were based on 100 replications.

The targeted FDR was chosen to be *α* = 0.10. Figs 2 and 3 summarized the empirical statistical power and false discovery proportion by six procedures based on 100 replications. It can be seen that the edge-count test was superior to Welch’s t test and mutual information test in both false discovery rate control and statistical power under all settings. Notably, the edge-count test showed a substantial power gain (ranging from 0.17 ∼ 0.44) over other tests. For independent features, the BH procedure and Efron’s procedure performs very similar in FDR control. However, under feature dependence, the BH procedure is slightly worse than Efron’s procedure for all tests.

**Fig 4:**
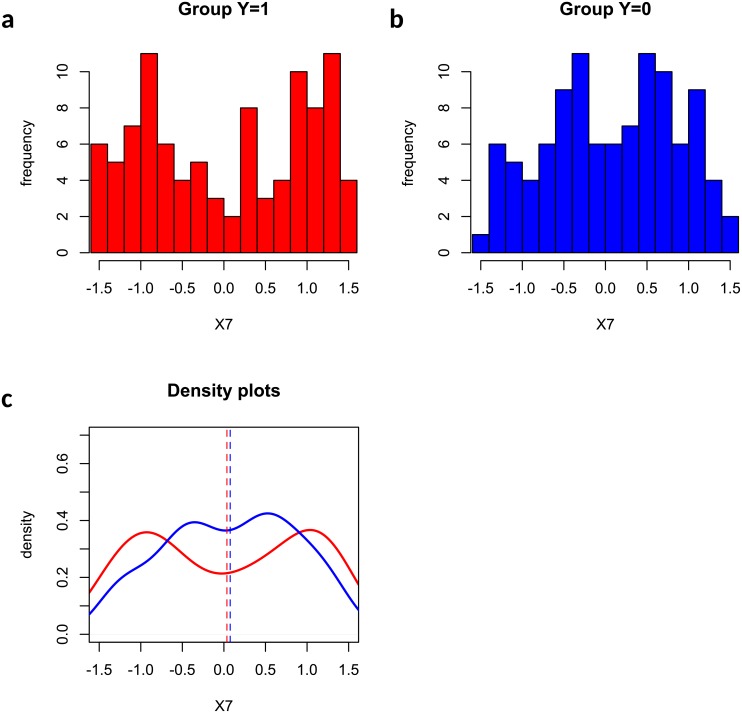
An example that feature *X*_7_ was captured by edge-count test but missed by Welch’s t test: (a) histogram of *X*_7_ in group *Y* = 1; (b) histogram of *X*_7_ in group *Y* = 0; (c) comparison of two fitted density curves, where the vertical dashed lines indicate the sample means in two groups.

**Fig 5:**
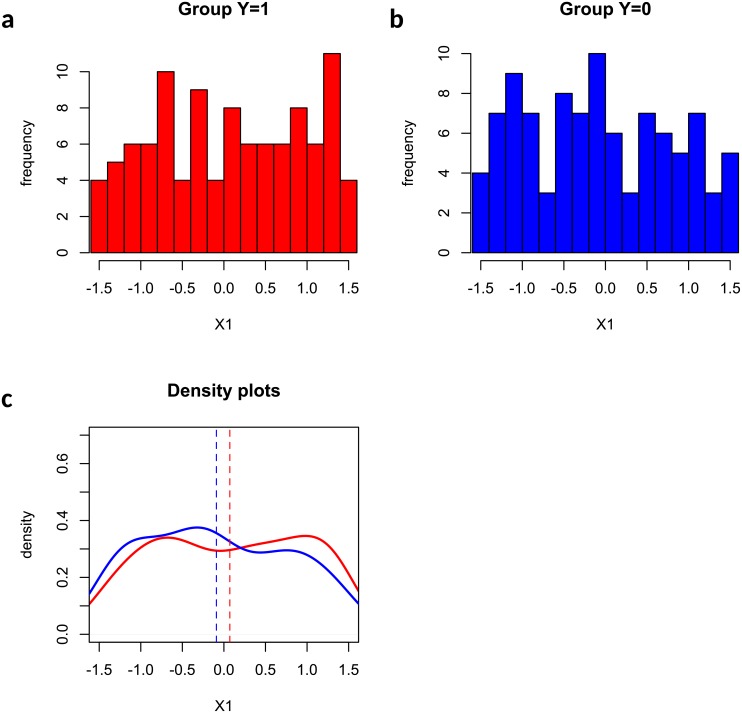
An example that feature *X*_7_ was missed by both edge-count test and Welch’s t test: (a) histogram of *X*_7_ in group *Y* = 1; (b) histogram of *X*_7_ in group *Y* = 0; (c) comparison of two fitted density curves, where the vertical dashed lines indicate the sample means in two groups.

Fig 4 presented an illustrative example where feature *X*_7_ was missed by Welch’s t test and mutual information test but captured by the edge-count test in our simulation. The reason is that feature *X*_7_ has a quadratic effect ($h_{7}\left(X_{7}\right)=X_{7}^{2}$) on *Y*, and the difference between two sample means (vertical dashed lines) becomes subtle and undetectable. However, feature *X*_7_ showed very different patterns in two groups (a clear bimodal shape in *Y* = 1 and much weaker bimodal shape in *Y* = 0) which was detected by the edge-count test. Fig 5 showed an example of false negative where the feature was missed by all methods due to a small difference in both sample mean and sample distributions.

### Application to three cancer genomic datasets

We first applied the new procedure to a colon cancer dataset [29] to search genes that differentiate cancer and normal subjects. The data contained expression level of 2,000 genes in 40 tumor and 22 colon tumor samples, probed by oligonucleotide arrays. To reduce variance and remove potential effects, the data for each subject were first log-transformed and then normalized by the trimmed mean and trimmed standard deviation (the lowest and highest 5% data were excluded). Two procedures were compared in selecting differentially expressed genes in two groups, including the edge-count method with Efron’s multiple testing procedure (a 3-MST was used as the similarity graph) and Welch’s t test with Benjamini-Hochberg procedure, both with targeted FDR *α* = 0.10. As can be seen from our simulation results (Figs 2 and 3), when the sample sizes are relatively small (*N* = 50), the mutual information z-test exhibited extremely low power, therefore we did not consider this method for real data analysis.

**Fig 6:**
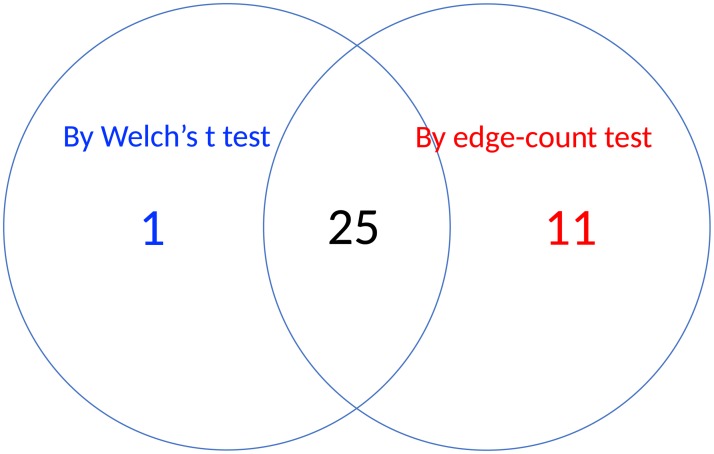
A Venn diagram showing the agreement between two selections by Welch’s t test (with BH procedure) and edge-count test (with Efron’s multiple testing procedure).

**Fig 7:**
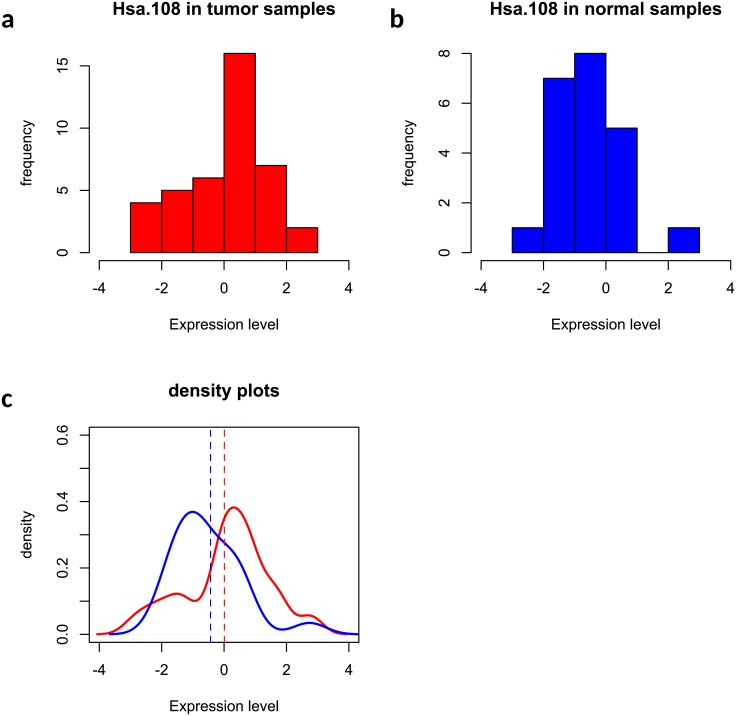
An example that gene *Hsa.108* was selected by edge-count test but missed by Welch’s t test: (a) histogram of gene *Hsa.108* in tumor samples; (b) histogram of gene *Hsa.108* in normal samples; (c) comparison of two fitted density curves, where the vertical dashed lines indicate the sample means in two phenotypic groups.

**Fig 8:**
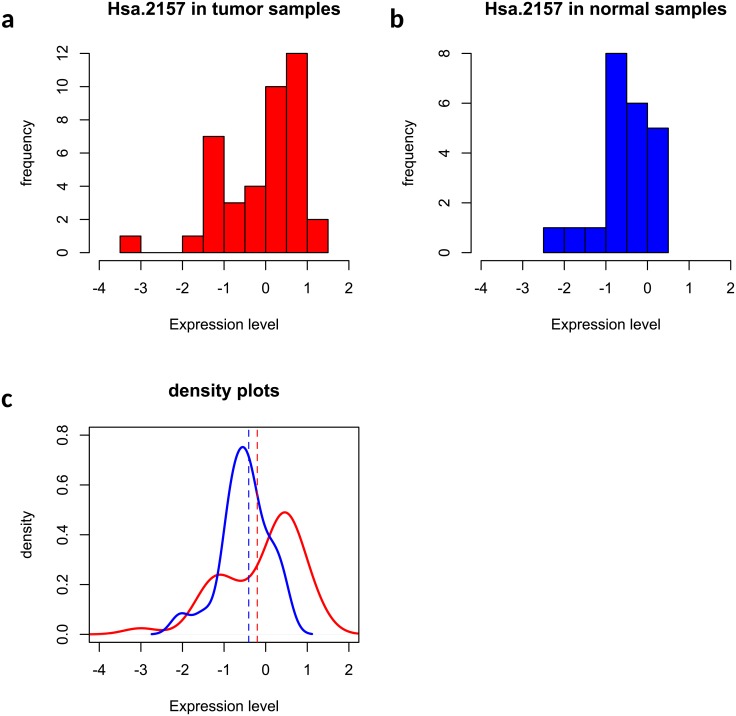
An example that gene *Hsa.2157* was selected by edge-count test but missed by Welch’s t test: (a) histogram of gene *Hsa.2157* in tumor samples; (b) histogram of gene *Hsa.2157* in normal samples; (c) comparison of two fitted density curves, where the vertical dashed lines indicate the sample means in two phenotypic groups.

Out of 2,000 genes, 36 and 26 genes were selected by the two methods and Fig 6 showed a Venn diagram summarizing the agreement between two selections. As shown in Fig 6, most of the 26 genes by Welch’s t test were also captured by the edge-count test, but a list of 11 genes that were identified by edge-count test were missed by the Welch’s t test, which included genes *Hsa.3180, Hsa.1804, Hsa.40177, Hsa.4937, Hsa.2157, Hsa.44676, Hsa.2847, Hsa.3026, Hsa.108, Hsa.11632, Hsa.27716*. Figs 7 and 8 presented the expression levels of two such genes, including *Hsa.108* and *Hsa.2157*, where the sample distributions in normal and tumor groups were significantly different from each other but both skewed. Our edge-count test successfully detected this difference, while the Welch’s t test failed to detect it due to close sample means (indicated by the two vertical dashed lines). Similar results were observed for the other nine genes (see Figs B-J in S1 File for details).

As previously reported in the literature, several of these 11 genes are associated with human cancers. To name a few, gene *Hsa.1804 (SFN)* promotes lung adenocarcinoma progression at an early stage [30]. Gene *Hsa.4937 (CREBBP)* acts as a potent tumor suppressor in small cell lung cancer, and inactivation of *CREBBP* enhances responses to a targeted therapy [31]. Gene *Hsa.44676 (VAV1)* promotes cancer growth by instigating tumor-microenvironment cross-talk via growth factor secretion [32]. Gene *Hsa.108 (POSTN)*, a matricellular protein-coding gene, has been shown to regulate key aspects of tumor biology, including proliferation, invasion, matrix remodeling, and dissemination to pre-metastatic niches in distant organs [33]. Gene *Hsa.11632 (RYR1)*, together with *RYR2* stimulates apoptosis of prostate cancer cells [34].

The results from colon cancer data well confirmed our findings from simulation study, i.e., the edge-count test can not only detect the mean difference, but also detect distributional differences, thus it is more sensitive to nonlinear change compared to normal-based tests such as t test, F test and Hotelling’s t test. Additionally, we conducted feature selection using p-values from a simple logistic regression (implemented by R function *glm()*), followed by a Benjamini-Hochberg procedure with *α* = 0.10. We detected a total of 28 significant genes, and 26 of them were consistent with the selection by Welch’s t test. However, this model fails to detect any of the 11 genes with nonlinear effects. The logistic regression model was further modified by adding a quadratic term in order to capture the nonlinear relations, however, this modification did not lead to any improvement.

The new method was further tested on two additional cancer genomic datasets, including the RNA-seq data for cervical cancer [35] and the microarray data for prostate cancer [36] (see S1 File for details about data analysis). Similar to the results from the colon cancer data, the edge-count test consistently detected more genes than the Welch’s t test (in the cervical cancer data, the new method identified 16 more genes and in the prostate cancer, the new method identified 12 more genes). All the newly discovered genes have close sample means but significantly different distributions in normal and tumor groups. The details of nine such genes were shown in S1 File, see Figs K-S in S1 File.

## Discussion

Genomic studies with high-dimensional data often rely on feature screening. In this work, we developed and validated a model-free feature screening method which reliably selects continuous features associated with a categorical outcome under high dimension. The new method tackles two major challenges in feature screening and feature selection, namely nonlinear effect detection and false discovery rate control under feature dependencies. The edge-count test is based on some simple calculations such as MST construction and Chi-square test, therefore it is easy-to-implement and feasible for large-scale data sets such as cancer genomic data and brain mapping data. For instance, in the colon cancer example with 2,000 genes, the computation took less than 10 seconds by R implementation on single CPU (2.5 GHz Intel Core i7).

There are several possible extensions of the proposed selector. For instance, in addition to feature screening, our method can also be used to select feature sets. One appealing property of the edge-count test is that it only requires a similarity graph constructed on the samples. In practice, one could simply build a MST or *m*-MST based on Euclidean distance as the similarity graph, and the main result $S_{i}\to \chi_{df=J}^{2}$ holds regardless of the sizes of feature sets. This extension can be used to search important pathways associated with certain disease, which is biologically more interesting than single gene based selection as the pathway-level analysis provides more functional insights into the mechanism underlying the phenotype change.

Efron’s multiple testing procedure was used in our method to control FDR under feature dependencies, but it might be replaced by other recently developed procedures. For instance, when the test statistics are positively dependent, one may also use Benjamini-Hochberg-Yekutieli (BHY) procedure to control FDR [37]. Fan et al. (2012) introduced a new multiple testing based on principal factor approximation, which adjusts the feature dependencies of arbitrary structure [38]. However, Fan et al.’s method relied on the true covariance matrix of the test statistics, which is unknown in most cases. To obtain a good sample covariance matrix of the test statistics {*S*_1_, …, *S*_*p*_} in our framework, a subsampling without replacement might be needed in order to get independent samples of {*S*_1_, …, *S*_*p*_}, however, the estimation may require relatively large sample size, e.g., *N* > 1, 000.

## Conclusions

Identification of disease-related biomarkers from large-scale data is essential in many genomic studies. However, existence of nonlinear effects and strong feature dependencies make existing methods inappropriate and unreliable. In this work, we presented a model-free feature screening method which is sensitive to both linear and nonlinear effects. In addition, the dependence-adjusted multiple testing procedure can well control the false discovery rate under feature dependencies. On a whole, we put forward a simple yet effective testing procedure that reliably captures different types of effects. Although we used gene expression data for illustration in the paper, the proposed test can be readily applied to other data types and problems, such as DNA methylation data and protein expression data and pathway selection.

## Supporting information

S1 File Additional analyses. This file contains additional simulation studies and real data applications, as well as the technical report by Zhang, Mahdi and Chen.
(PDF)

## Abbreviations

BH: Benjamini-Hochberg
BHY: Benjamini-Hochberg-Yekutieli
FDR: False discovery rate
MST: Minimum spanning tree
