# Supplementary material for: Model-free feature screening for categorical outcomes: Nonlinear effect detection and false discovery rate control
Source: PLoS One. 2019 May 31;14(5):e0217463. doi: 10.1371/journal.pone.0217463 (PMC6544247; doi:10.1371/journal.pone.0217463)
Supplement: S1 File — This file contains additional simulation studies and real data applications, as well as the technical report by Zhang, Mahdi and Chen. (PDF) [file pone.0217463.s001.pdf]

S1 File for "Model-free feature screening for categorical outcomes: nonlinear effect detection and false discovery rate control"

Qingyang Zhang[1*], Yuchun Du[2*]

[1]Department of Mathematical Sciences, University of Arkansas, AR, USA

[2]Department of Biological Sciences, University of Arkansas, AR, USA

* qz008@uark.edu, ydu@uark.edu

A. Simulation study on Kolmogorov-Smirnov test

A simulation study was conducted to illustrate the conservativeness of two-sample KS test. In particular, we compared the KS test and the new edge-count test in the following settings:

- Setting S1 (Uniform versus Gaussian): $X_i \sim \text{Uniform}(0,2)$, $Y_j \sim \text{N}(1,\frac{1}{3}), i = 1,2,...,n; j = 1,2,...,n$
- Setting S2 (Gaussian versus Gaussian mixture): $X_i \sim \text{N}(1,1.1), Y_j \sim \frac{1}{2}\text{N}(0,\frac{1}{2})+\frac{1}{2}\text{N}(2,\frac{1}{2}), i = 1,2,...,n; j = 1,2,...,n$

For both settings, sample sizes $n = 50,100,200,300,500$ were used and the performance of the two methods were evaluated based on 1,000 simulations. For edge-count test, a 3-MST was used as the similarity graph and p-value was evaluated by the asymptotic Chi-squared distribution. S1 Fig summarized the empirical statistical power (proportion of correct rejections) by two methods, where it can be seen that the KS test

is rather conservative even for relatively large sample size. For instance, with 300 samples in each group, the KS test exhibited an extremely low statistical power (below 20%) while the edge-count test achieved a satisfactory power (70∼80%) in both settings. Table A in S1 File showed the computing times of two tests in 1,000 simulations. The edge-count test is substantially more time-consuming than KS test due to several intermediate steps such as the calculation of minimum spanning trees.

B. Details of the other nine genes identified by edge-count test only

Figures B-J showed the distributions of the other nine genes detected by edge-count test by missed by Welch's t test, including *Hsa.3180, Hsa.1804, Hsa.40177, Hsa.4937, Hsa.44676, Hsa.2847, Hsa.3026, Hsa.11632, Hsa.27716*, in normal and colon cancer subjects.

C. Application to cervical and prostate cancer data

The new method was further tested on two additional datasets, namely the RNA-seq data for cervical cancer [1] and the microarray data for prostate cancer [2]. The cervical cancer data contains the expression levels of 714 microRNAs on 58 samples (29 tumor samples and 29 normal controls). The prostate cancer data contains the expression levels (microarray) of 2,000 genes (preselected from a total of 26,000 genes) on 103 samples consisted of 41 normal samples and 62 tumor samples. For the RNA-seq data, the counts were transformed by $\log_2(x+1)$ and same normalization was applied to the transformed data as we did for the colon cancer data. Two methods including (1) Welch's t test with BH procedure and (2) edge-count test with Efron's procedure were applied to both data sets, with FDR $\alpha = 0.10$.

Similar as in the colon cancer data, the edge-count test with Efron's procedure consistently detected

more genes than the Welch's t test. For instance, in the cervical cancer data, 71 genes were detected by our new method while only 55 genes were detected by the Welch's t test. In the prostate cancer, the new method identified 42 genes while the Welch's t test only detected 30 genes. All the newly discovered genes (identified by edge-count test but missed by Welch's t test) have similar sample means but significantly different distributions in normal and tumor groups. The details of nine such genes/miRNAs, including *miR-17, miR-30c-2, miR-329, miR-433, HEXB, PXR1, MTCP1, CAMK1D, ATP6V1G1*, were shown in Figure K-S in S1 File.

D. Finite sample performance of the asymptotic null distribution

We conducted a simulation study to evaluate the finite sample performance of the asymptotic chi-squared distribution. We fixed the number of groups at $J = 4$ and generated data under two models (1) $X|Y = j \sim N(0,1), j = 1,2,3,4$ (2) $X|Y = j \sim \exp(1), j = 1,2,3,4$. For each model, we used three different sample sizes (1) $n_1 = n_2 = n_3 = n_4 = 20$ (2) $n_1 = n_2 = 20, n_3 = n_4 = 50$ (3) $n_1 = n_2 = n_3 = n_4 = 50$. In addition, we compared three different similarity graphs (1) 1-MST (2) 3-MST (3) 5-MST. For each generated dataset, we computed two p-values, including the asymptotic p-value from chi-squared distribution and the p-value based on 10,000 permutations. The difference between these two p-values based on 100 simulations were summarized using boxplots in Figure T in S1 File.

References

[1] Witten, D., Tibshirani, R., Gu, S., Fire, A., Lui, W. (2010), Ultra-high throughput sequencing-based small RNA discovery and discrete statistical biomarker analysis in a collection of cervical tumours and matched controls, *BMC Biology*, **8**(58)

[2] Lapointe, J., Li, C., Higgins, J., van de Rijn, M., Bair, E. et al. (2004), Gene expression profiling identifies clinically relevant subtypes of prostate cancer, *Proceedings of the National Academy of Science of the United States of America*, **101**(3), 811-6.

Tables and Figures

Table A: Computing time of KS test and edge-count test

Sample size	KS test	Edge-count test
50	1.50	4.77
100	1.57	7.89
200	1.68	10.90
300	1.82	13.67
500	2.32	18.06

Presented in the table are the CPU time (in seconds) by KS test and edge-count test for 1000 simulations.

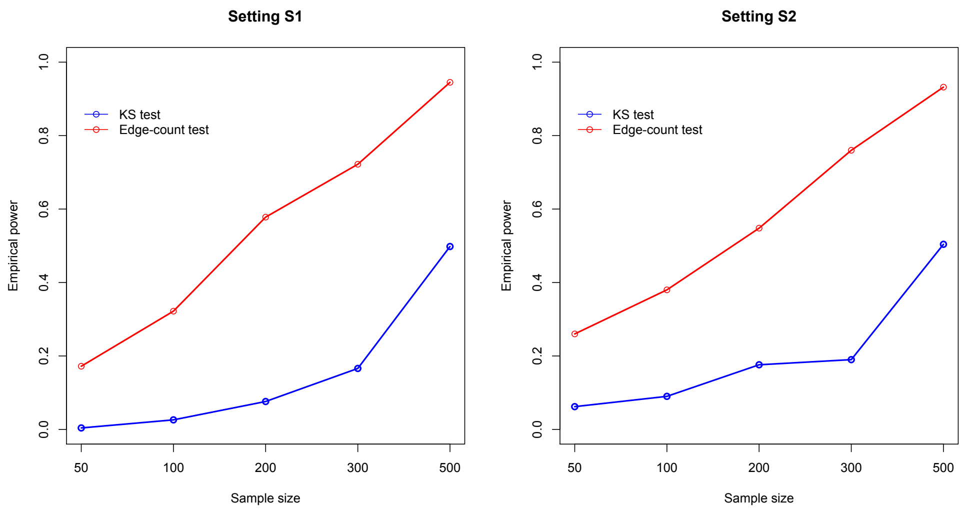

Figure A: Power comparison between Kolmogorov-Smirnov test and edge-count test.

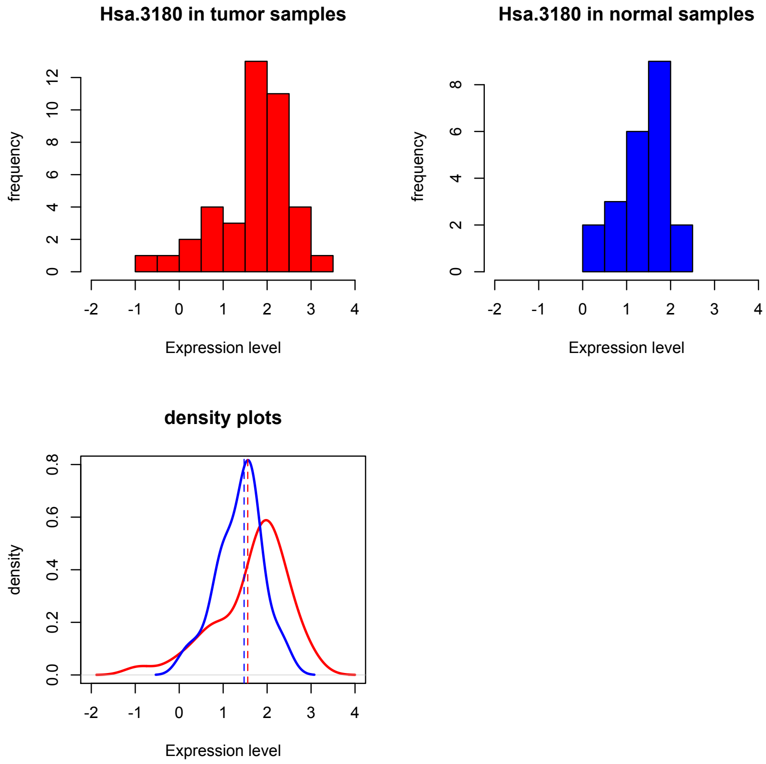

Figure B: An example that gene Hsa.3180 was selected by edge-count test but missed by Welch's t test: (a) histogram of gene Hsa.3180 in tumor samples; (b) histogram of gene Hsa.3180 in normal samples; (c) comparison of two fitted density curves, where the vertical dashed lines indicate the sample means in two phenotypic groups.

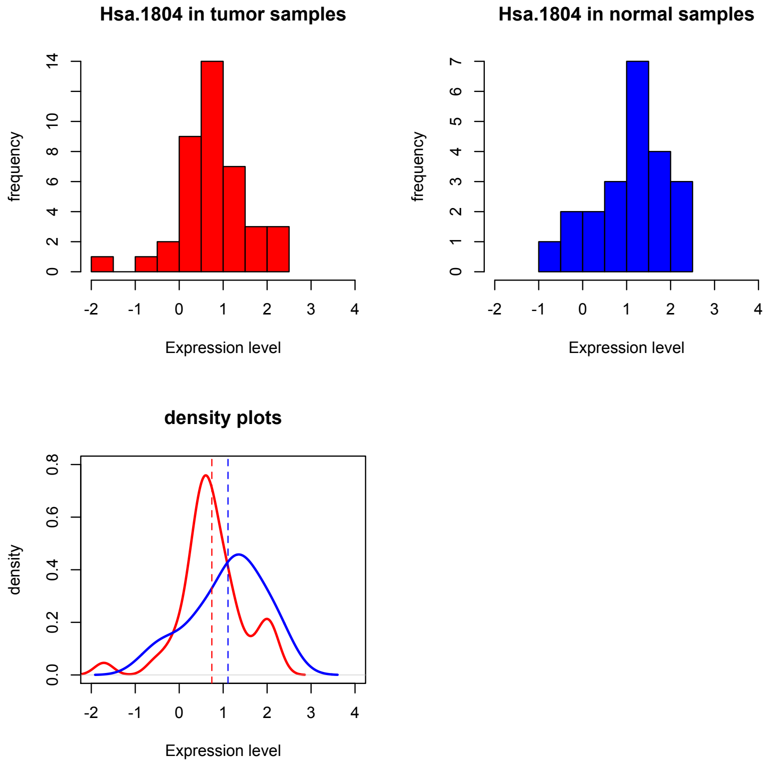

Figure C: An example that gene Hsa.1084 was selected by edge-count test but missed by Welch's t test: (a) histogram of gene Hsa.1084 in tumor samples; (b) histogram of gene Hsa.1084 in normal samples; (c) comparison of two fitted density curves, where the vertical dashed lines indicate the sample means in two phenotypic groups.

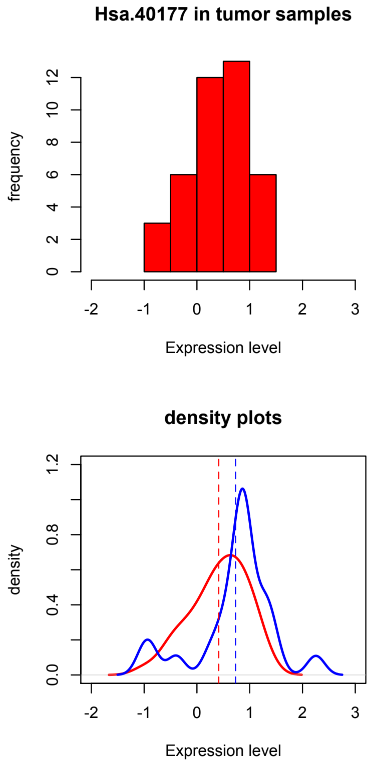

Figure D: An example that gene Hsa.40177 was selected by edge-count test but missed by Welch's t test: (a) histogram of gene Hsa.40177 in tumor samples; (b) histogram of gene Hsa.40177 in normal samples; (c) comparison of two fitted density curves, where the vertical dashed lines indicate the sample means in two phenotypic groups.

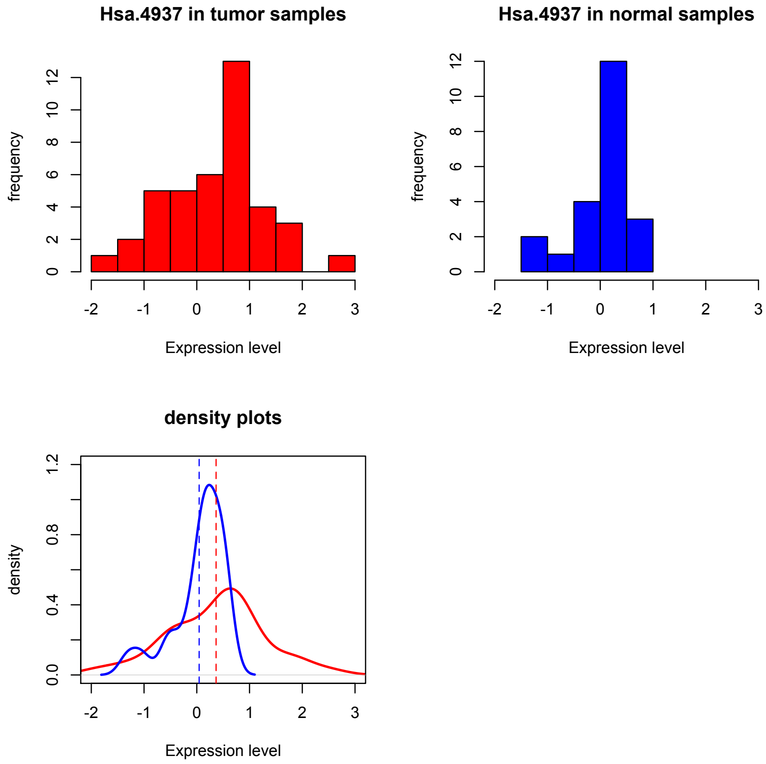

Figure E: An example that gene Hsa.4937 was selected by edge-count test but missed by Welch's t test: (a) histogram of gene Hsa.4937 in tumor samples; (b) histogram of gene Hsa.4937 in normal samples; (c) comparison of two fitted density curves, where the vertical dashed lines indicate the sample means in two phenotypic groups.

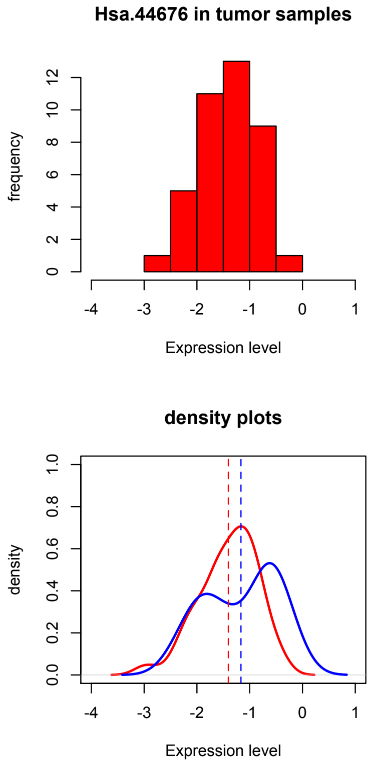

Figure F: An example that gene Hsa.44676 was selected by edge-count test but missed by Welch's t test: (a) histogram of gene Hsa.44676 in tumor samples; (b) histogram of gene Hsa.44676 in normal samples; (c) comparison of two fitted density curves, where the vertical dashed lines indicate the sample means in two phenotypic groups.

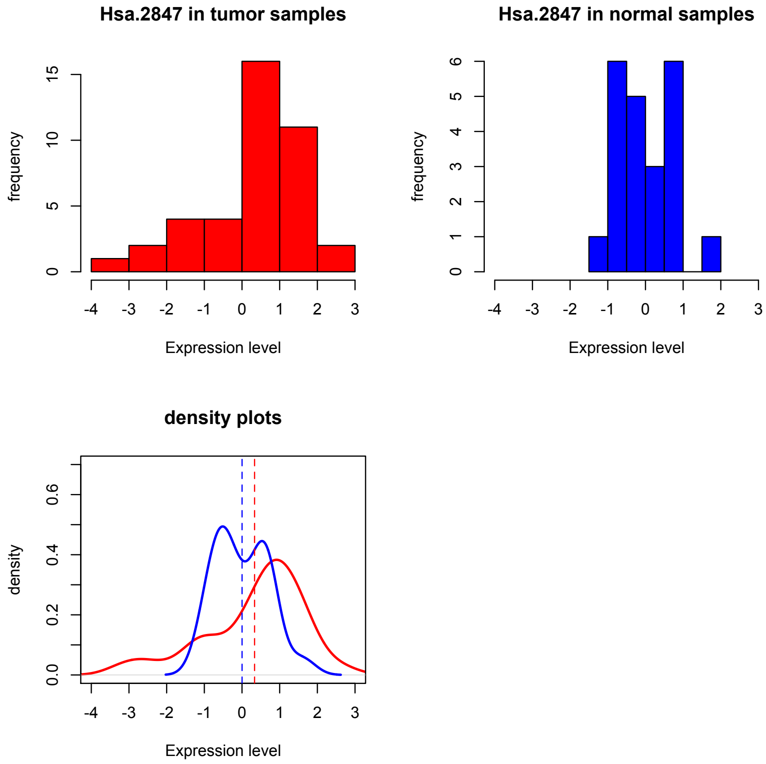

Figure G: An example that gene Hsa.2847 was selected by edge-count test but missed by Welch's t test: (a) histogram of gene Hsa.2847 in tumor samples; (b) histogram of gene Hsa.2847 in normal samples; (c) comparison of two fitted density curves, where the vertical dashed lines indicate the sample means in two phenotypic groups.

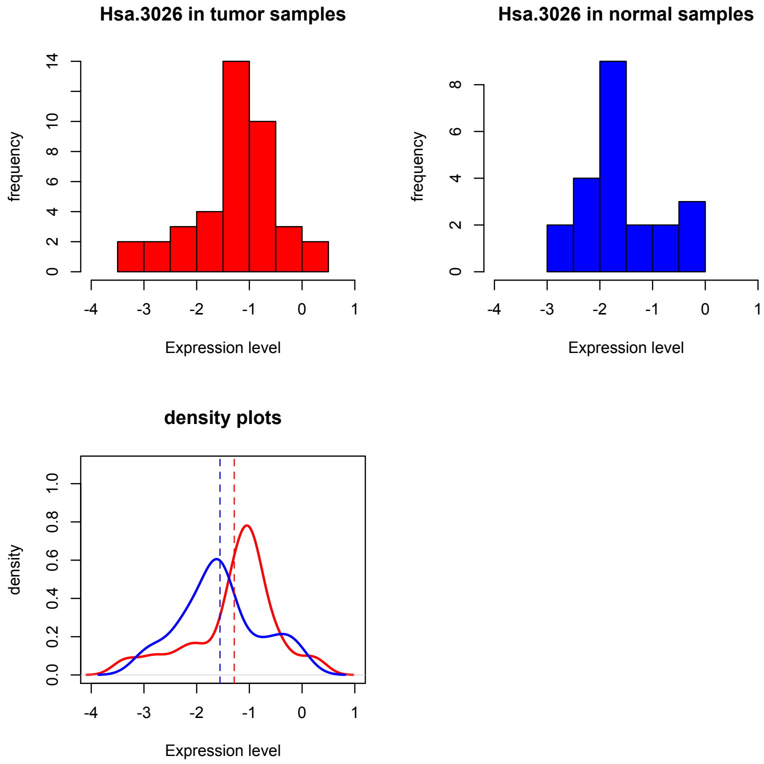

Figure H: An example that gene Hsa.3026 was selected by edge-count test but missed by Welch's t test: (a) histogram of gene Hsa.3026 in tumor samples; (b) histogram of gene Hsa.3026 in normal samples; (c) comparison of two fitted density curves, where the vertical dashed lines indicate the sample means in two phenotypic groups.

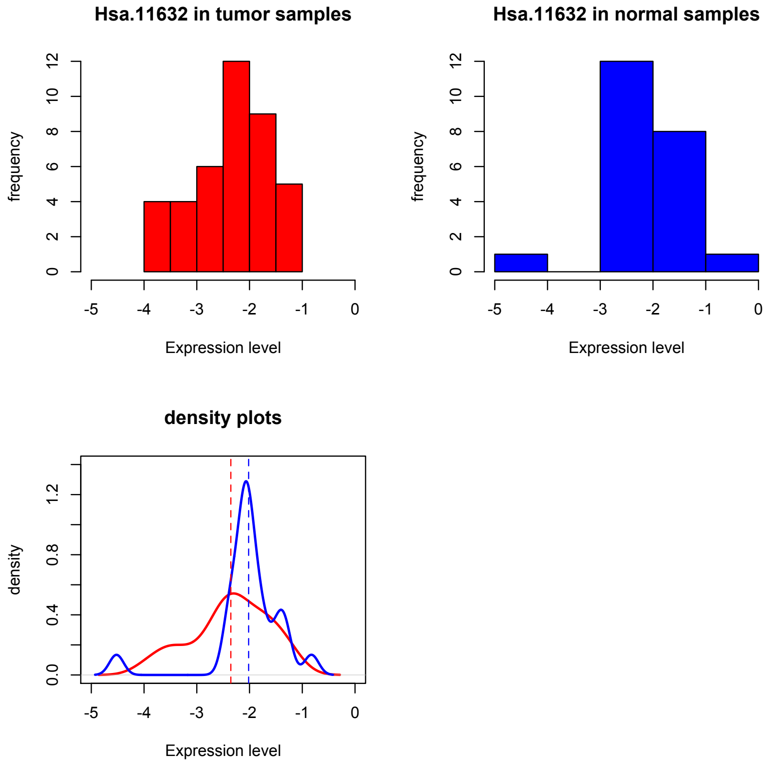

Figure I: An example that gene Hsa.11632 was selected by edge-count test but missed by Welch's t test: (a) histogram of gene Hsa.11632 in tumor samples; (b) histogram of gene Hsa.11632 in normal samples; (c) comparison of two fitted density curves, where the vertical dashed lines indicate the sample means in two phenotypic groups.

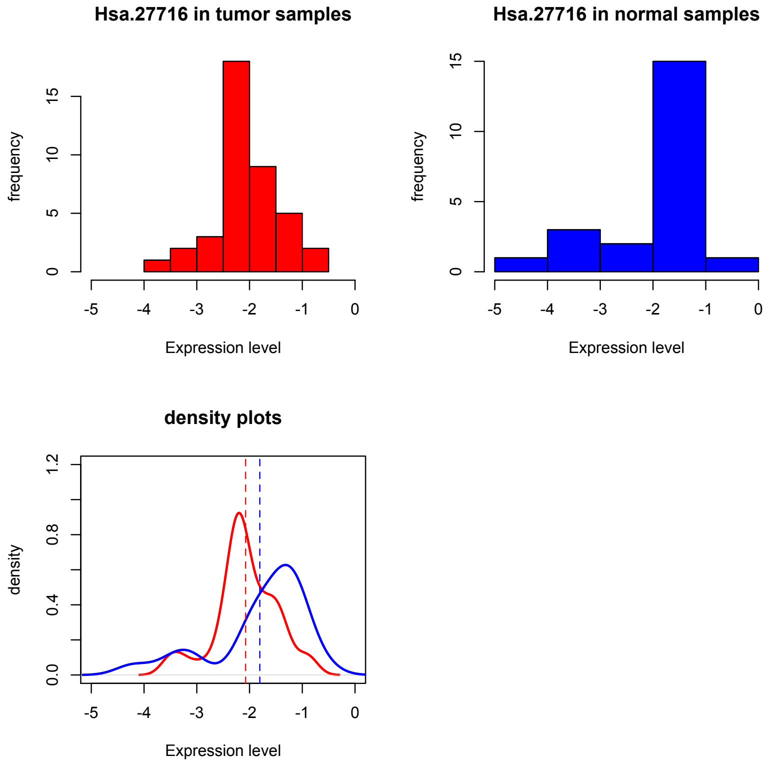

Figure J: An example that gene Hsa.27716 was selected by edge-count test but missed by Welch's t test: (a) histogram of gene Hsa.27716 in tumor samples; (b) histogram of gene Hsa.27716 in normal samples; (c) comparison of two fitted density curves, where the vertical dashed lines indicate the sample means in two phenotypic groups.

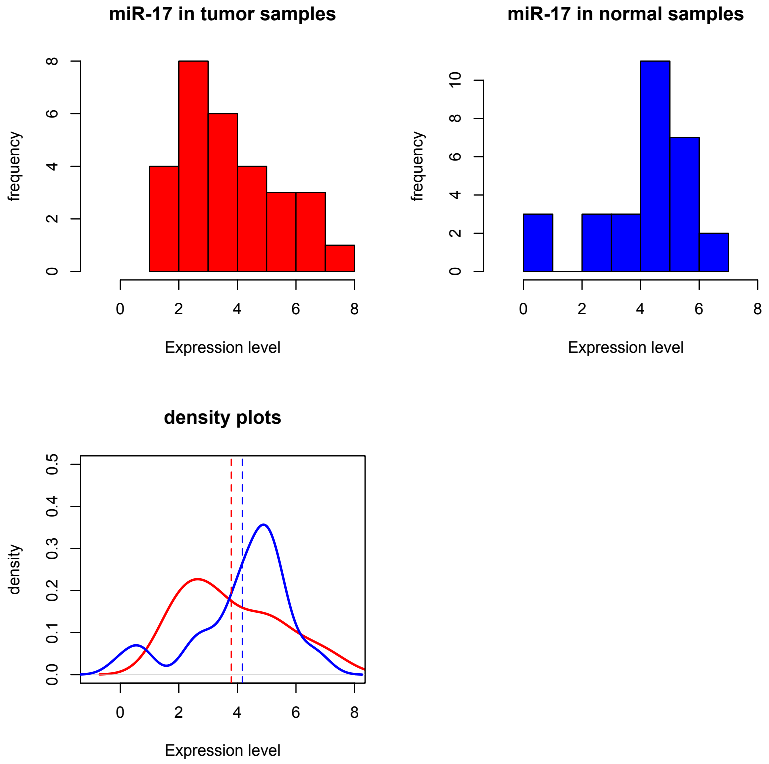

Figure K: An example that gene miR-17 was selected by edge-count test but missed by Welch's t test: (a) histogram of gene miR-17 in tumor samples; (b) histogram of gene miR-17 in normal samples; (c) comparison of two fitted density curves, where the vertical dashed lines indicate the sample means in two phenotypic groups.

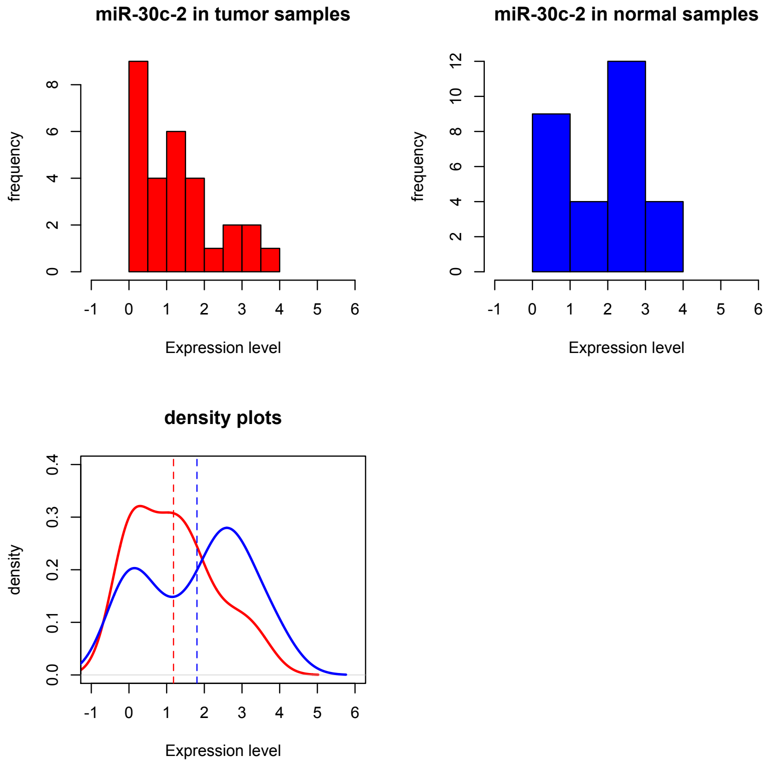

Figure L: An example that gene miR-30c-2 was selected by edge-count test but missed by Welch's t test: (a) histogram of gene miR-30c-2 in tumor samples; (b) histogram of gene miR-30c-2 in normal samples; (c) comparison of two fitted density curves, where the vertical dashed lines indicate the sample means in two phenotypic groups.

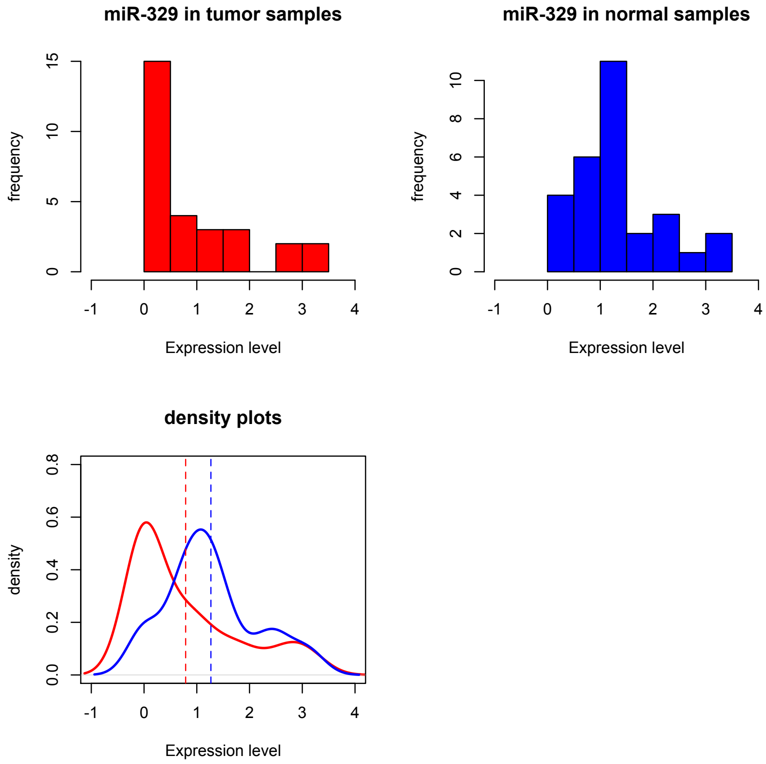

Figure M: An example that gene miR-329 was selected by edge-count test but missed by Welch's t test: (a) histogram of gene miR-329 in tumor samples; (b) histogram of gene miR-329 in normal samples; (c) comparison of two fitted density curves, where the vertical dashed lines indicate the sample means in two phenotypic groups.

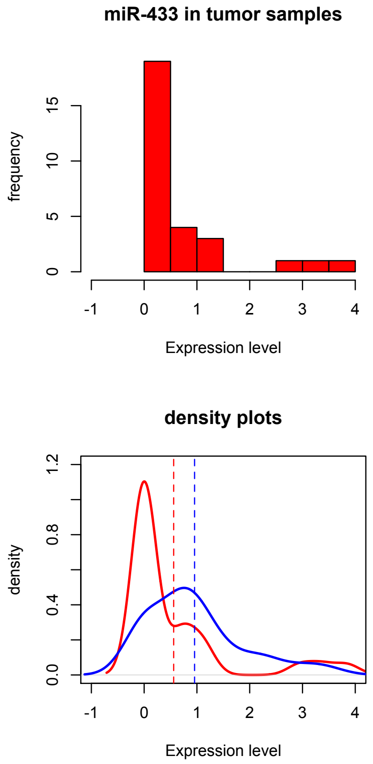

Figure N: An example that gene miR-433 was selected by edge-count test but missed by Welch's t test: (a) histogram of gene miR-433 in tumor samples; (b) histogram of gene miR-433 in normal samples; (c) comparison of two fitted density curves, where the vertical dashed lines indicate the sample means in two phenotypic groups.

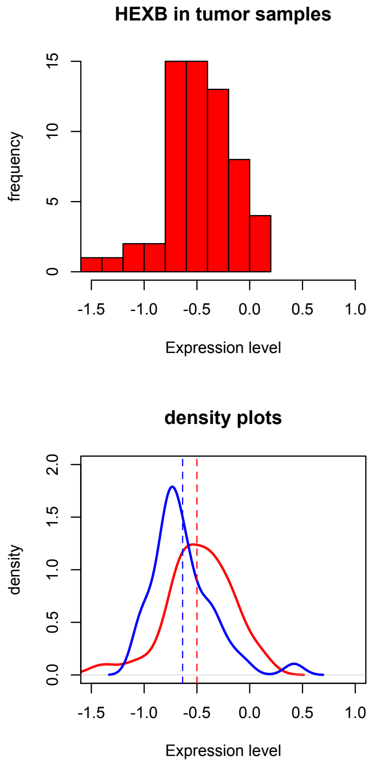

Figure O: An example that gene HEXB was selected by edge-count test but missed by Welch's t test: (a) histogram of gene HEXB in tumor samples; (b) histogram of gene HEXB in normal samples; (c) comparison of two fitted density curves, where the vertical dashed lines indicate the sample means in two phenotypic groups.

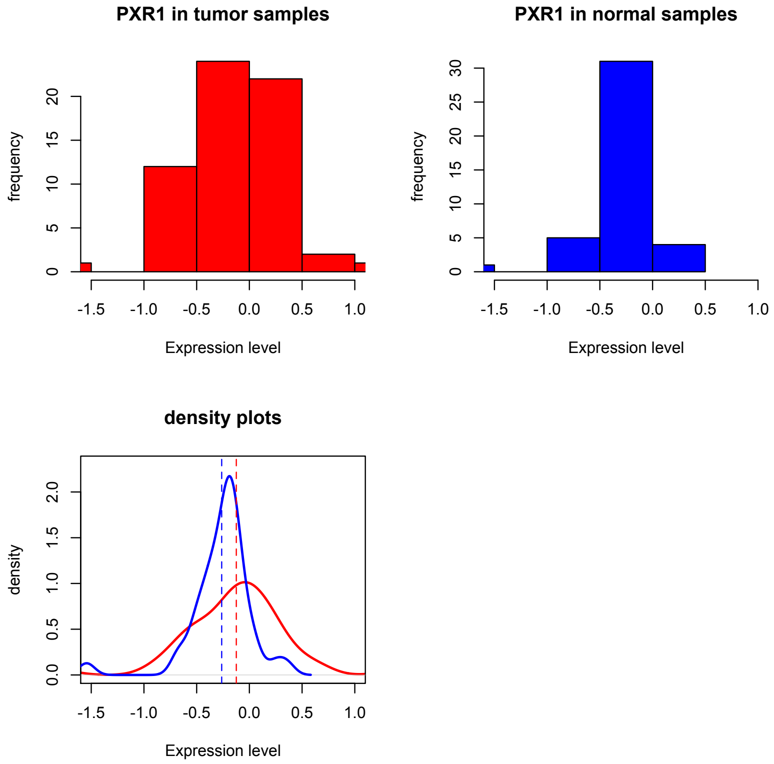

Figure P: An example that gene PXR1 was selected by edge-count test but missed by Welch's t test: (a) histogram of gene PXR1 in tumor samples; (b) histogram of gene PXR1 in normal samples; (c) comparison of two fitted density curves, where the vertical dashed lines indicate the sample means in two phenotypic groups.

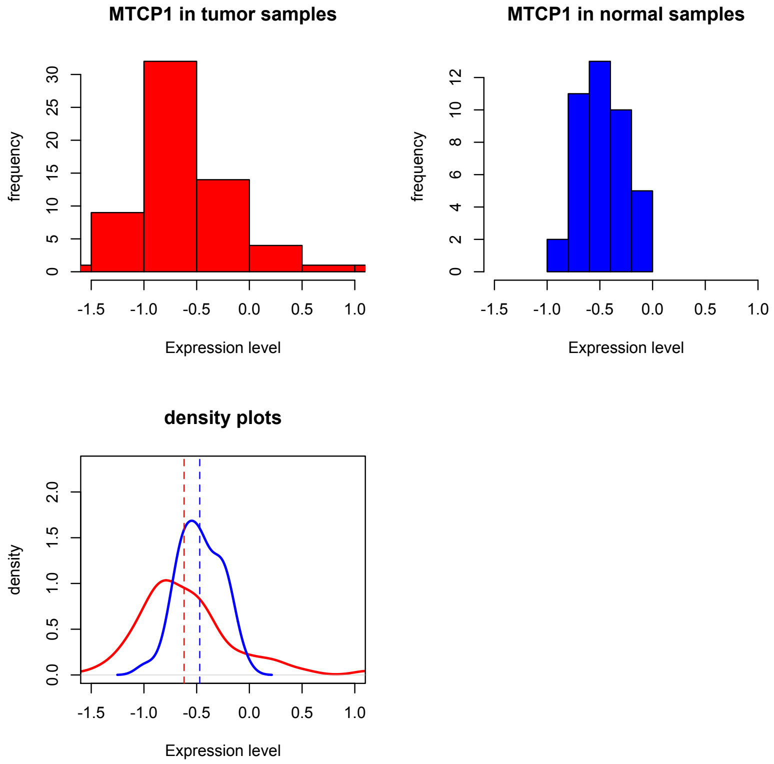

Figure Q: An example that gene MTCP1 was selected by edge-count test but missed by Welch's t test: (a) histogram of gene MTCP1 in tumor samples; (b) histogram of gene MTCP1 in normal samples; (c) comparison of two fitted density curves, where the vertical dashed lines indicate the sample means in two phenotypic groups.

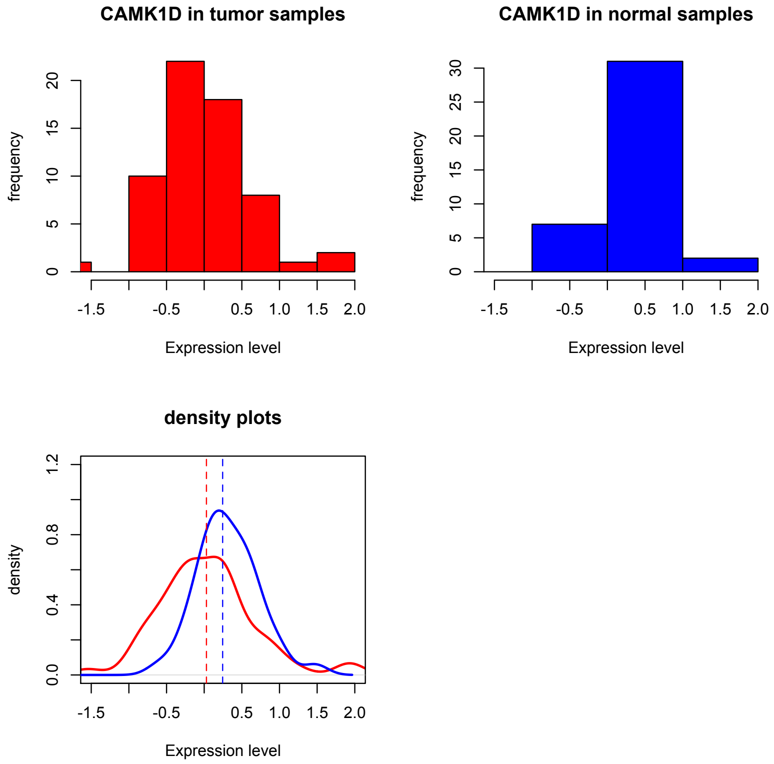

Figure R: An example that gene CAMK1D was selected by edge-count test but missed by Welch's t test: (a) histogram of gene CAMK1D in tumor samples; (b) histogram of gene CAMK1D in normal samples; (c) comparison of two fitted density curves, where the vertical dashed lines indicate the sample means in two phenotypic groups.

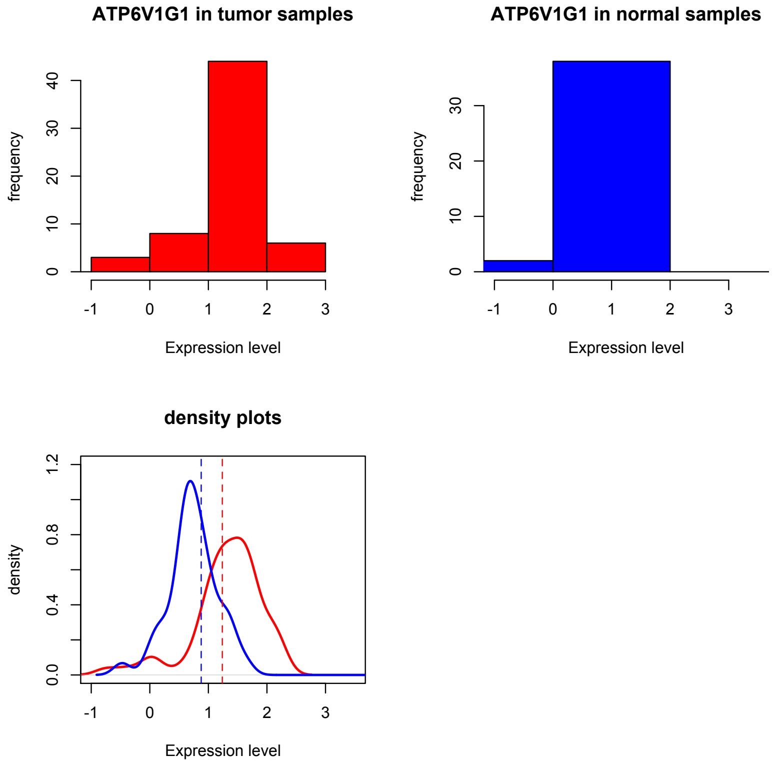

Figure S: An example that gene ATP6V1G1 was selected by edge-count test but missed by Welch's t test: (a) histogram of gene ATP6V1G1 in tumor samples; (b) histogram of gene ATP6V1G1 in normal samples; (c) comparison of two fitted density curves, where the vertical dashed lines indicate the sample means in two phenotypic groups.

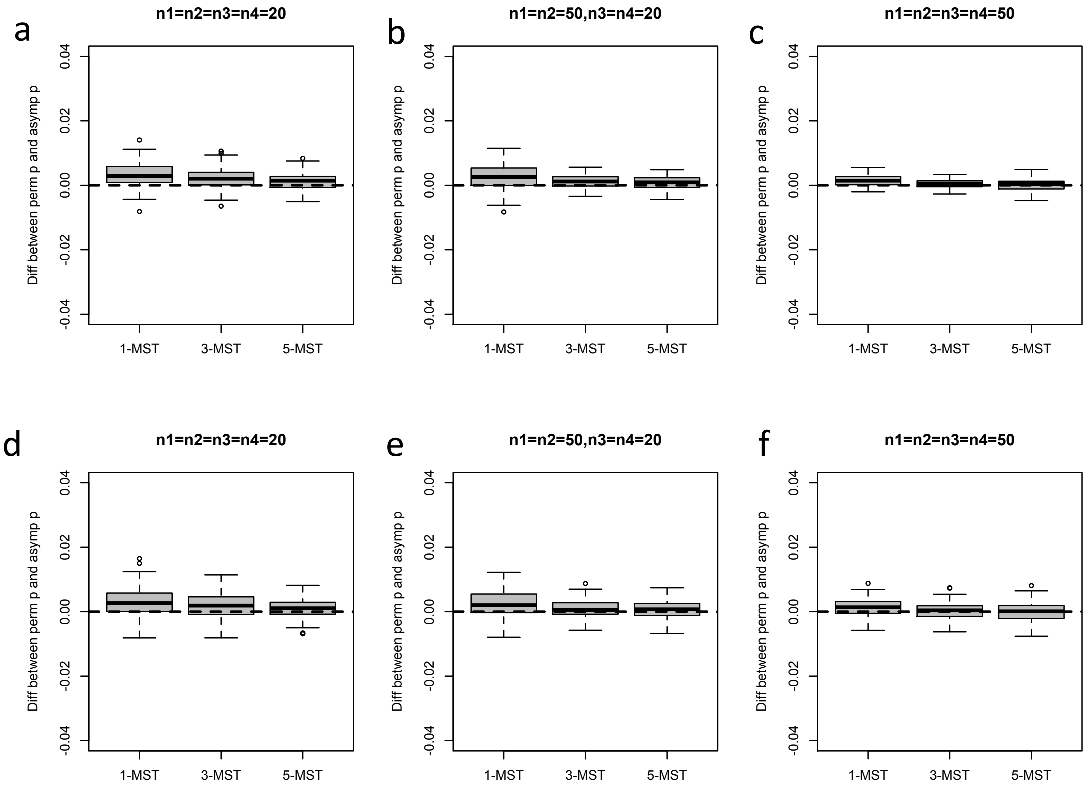

Figure T: Boxplots of the difference between approximate p-value and permutation p-value (approximate p minus permutation p), under different sample sizes, similarity graphs (1-MST, 3-MST and 5-MST), and two models (a, b, c for Gaussian model $N(0,1)$ and d, e, f for exponential model $exp(1)$)

A Graph-Based Multi-Sample Test for Identifying Pathways Associated with Cancer Progression

Qingyang Zhang[1], Ghadeer Mahdi[1], Hao Chen[2]

[1]Department of Mathematical Sciences, University of Arkansas, AR 72701

[2]Department of Statistics, University of California at Davis, CA 95616

All authors contributed equally to this work

Correspondence should be addressed to: qz008@uark.edu, hxchen@ucdavis.edu

Abstract

Background: Cancer is a complex disease in the sense that it is not a result of an abnormality of a single gene but a consequence of changes in many genes, it is therefore of great importance to understand the roles of different oncogenic and tumor suppressor pathways in tumorigenesis. In recent years, there have been many computational models developed to study the genetic alterations of different pathways in the evolutionary process of cancer. However, most of the methods are knowledge-based enrichment analyses and inflexible to analyze user-defined pathways or gene sets.

Results: In this paper, we develop a nonparametric and data-driven approach to testing for the dynamic changes of pathways over the cancer progression. Our method is based on an expansion and refinement of the pathway being studied, followed by a graph-based multivariate test, which is very easy to implement in practice. The new test is applied to the rich Cancer Genome Atlas data to study the (epi)genetic alterations of 186 KEGG pathways in the development of serous ovarian cancer. To make use of the comprehensive data, we incorporate three data types in the analysis including gene expression level, copy number and DNA methylation level. Our analysis suggests a list of nine pathways, including cell cycle, ERBB, JAK-STAT signaling and p53 signaling pathways, that are closely associated with serous ovarian cancer progression. By pairwise tests, it is found that most of the identified pathways contribute only to a particular transition step. For instance, the cell cycle and ERBB pathways play key roles in the early-stage transition, while the ECM receptor and apoptosis pathways contribute to the progression from stage III to stage IV.

Conclusions: The proposed computational pipeline is powerful in detecting important pathways or gene

sets that drive cancers at certain stage(s), thus may offer new insights into the understanding of molecular mechanism of cancer initiation and progression.

Keywords: edge-count test; tumorigenesis; serous ovarian cancer; pathway analysis; the Cancer Genome Atlas;

1 Background

Cancer is a heterogeneous disease driven by abnormality of multiple genetic or epigenetic factors, such as gene expression level, DNA methylation level, somatic mutation and copy number variation. Over the past decades, many individual genes have been discovered to govern important functions in different cancer types, for instance, genes *BRCA1*, *BRCA2*, *PIK3C* and *GATA3* for breast cancer (TCGA Research Network. [2012]), genes *MYC*, *RIT1*, *ECFR* and *ERBB2* for prostate cancer (TCGA Research Network. [2014]). These individual genes were usually identified through univariate two-sample comparison between normal and tumor groups. Although the single-gene analyses are powerful in detecting important oncogenes and suppressors as potential treatment targets, they provided very limited insights into the molecular mechanisms of tumorigenesis, as it omitted all regulatory relations between genes (Hoek et al. [2006]; Ivshina et al. [2006]; Ma et al. [2003]; Pancione et al. [2012]; Smith et al. [2005]; Subramanian et al. [2005]; Talantov et al. [2005]; Tomlins et al. [2007]). From the view of systems biology, a simultaneous analysis of a gene set or a genetic pathway might provide more clear functional insight into cause of the phenotypic changes. There have been many recently developed tools for gene set analysis, including EnrichNet (Glaab et al. [2012]), GAGE (Luo et al. [2009]), CSEA (Al-Shahrour et al. [2007]), PAGE (Kim and Volsky [2005]), MEGO (Tu et al. [2005]), Catmap (Breslin et al. [2004]), ErmineJ (Lee et al. [2005]), and GeneTrail (Backes et al. [2007]). Most of these approaches, however, are knowledge-based and inflexible to user-defined pathways. One exception is Edelman et al. [2008], which introduced a data-driven approach to modeling cancer progression via pathway dependencies and flexible to any user-defined pathways.

Unlike the knowledge-based enrichment analysis, Edelman et al.'s method is essentially a hierarchical analysis that targets pathways relevant to particular transition between cancer stages, for instance, from normal to primary tumor, and from primary tumor to metastasis. However, Edelman's approach relied on several computationally intensive steps, such as regularized multi-task learning, inverse regression, learning

gradients as well as leave-one-out cross validation, which greatly limited its application to large-scale data. To this end, we proposed an efficient and powerful test based on inter-point distance to identify cancer-driving pathways. Similar as Edelman's method, our method is purely data-driven and capable of dealing with new pathways adapted to certain tissue/disease. In the meanwhile, the test is model-free and very easy to implement as it only requires few simple calculations, such as minimum spanning tree and Chi-square test.

The rest of the paper is structured as follows: In Section 2, we introduce the new test and establish its asymptotic distribution. The finite sample performance is evaluated via simulations. In Section 3, we apply the new test to the rich Cancer Genome Atlas data to identify important pathways that drive serous ovarian cancer. We discuss the strengths and shortcomings of our approach in Section 4 and conclude this paper in Section 5. Technical proof about the asymptotic distribution of the edge-count test is provided in the Appendix.

2 Methods

2.1 Problem formulation

Detecting cancer-driving pathways is essentially detecting differentially acted pathways between cancer stages. Here, we formulate it as a statistical problem of testing the equality of two or multiple joint distributions, where each random variable represents the expression level of one gene. To be precise, we let $i \in \{1,2,...,p\}$ be the index for cancer stages, and $(X_1^{(i)},...,X_d^{(i)})$ be the expression levels of d genes in the pathway being studied, with a joint distribution $\boldsymbol{F}^{(i)}$. Given n_i i.i.d. observations in stage i, $(\mathbf{x}_1^{(i)},\mathbf{x}_2^{(i)},...,\mathbf{x}_{n_i}^{(i)})$, where $\mathbf{x}_k^{(i)} = (x_{k1}^{(i)},x_{k2}^{(i)},...,x_{kd}^{(i)})$, we concern the following hypothesis testing:

$$
\begin{aligned}
H_0: &\ \boldsymbol{F}^{(1)} = \boldsymbol{F}^{(2)} = ... = \boldsymbol{F}^{(p)}, \\
H_\alpha: &\ \boldsymbol{F}^{(j)} \neq \boldsymbol{F}^{(j')}, \text{for some } \ j, j', 1 \leq j, j' \leq p.
\end{aligned}
$$

For a particular transition step, from stage i to stage $i+1$ ($i = 1, ..., p-1$), we could conduct the following pairwise test, which is a special case when $p = 2$

$$H_0: \ \boldsymbol{F}^{(i)} = \boldsymbol{F}^{(i+1)},$$
$$H_\alpha: \ \boldsymbol{F}^{(i)} \neq \boldsymbol{F}^{(i+1)}.$$

2.2 Graph-based multivariate test

The two-sample multivariate tests in the statistics literature can be roughly classified into two categories, namely the parametric and nonparametric multivariate tests. Hotelling's T is a simple and widely used test for comparing the mean vectors of two independent populations, which generally works well for low-dimension case. There are also numerous tests recently developed for high dimensions (Cai et al. [2014]; Gregory et al. [2015]; Srivastava et al. [2013]), which have mostly focused on the equality of mean vectors. Unlike the parametric tests, the nonparametric tests directly test the equality of two multivariate distributions, for instance, Kolmogorov-Smirnov (KS) test (Lopes et al. [2008]) and graph-based test (Friedman and Rafsky [1979]; Rosenbaum [2005]), which are two popular families of such test, but both have practical limitations in real world applications. KS test is known to be very conservative, i.e., the null hypothesis is too often not rejected. Moreover, by the brute force algorithm, the implementation of multi-dimensional KS test can be prohibitively computationally intensive. Graph-based tests such as edge-count tests are easy to implement but they could be problematic under certain location and scale alternatives, as pointed out by Chen and Friedman [2017]. The rationale of the edge count test is that if two groups have different distributions, samples would be preferentially closer to others from the same group than those from the other group in a similarity graph, therefore the edges in the graph would be more likely to connect samples from the same group. The test rejects the null if the number of between-group edges is significantly less than expected. Chen and Friedman [2017] developed an modified edge-count test and established its asymptotic distribution under two samples. This test works properly under different alternatives and exhibits substantial power gains over existing edge-count tests. Similar as other edge-count tests, their test is based upon a similarity graph constructed over the pooled samples from different groups. For instance, one could use a minimum spanning tree (MST, Cheriton and Tarjan [2006]) based on euclidean distance as the similarity graph.

In order to test the hypotheses in 2.1, we extended Chen and Friedman's test to a multi-sample case and

derived the asymptotic result for p-value approximation. We began with pooling samples from all p groups and indexing them by $1,2,...,N=\sum_{i=1}^{p} n_i$. A similarity graph, denoted by G, was then constructed on the pooled observations. Let R_i be the number of edges in the graph that connect observations within sample i. We worked under the permutation null distribution, which places $1/\binom{N}{n_1,n_2,...,n_p}$ probability on each of the $\binom{N}{n_1,n_2,...,n_p}$ choices of n_i out of the total N observations as sample i, $i=1,\dots,p$, with each observation chosen once. When there is no further specification, we denote by P_P, E_P, Var_P, Cov_P probability, expectation, variance, and covariance, respectively, under the permutation null distribution.

It is not hard to show that:

$$\mathsf{E}_\mathsf{P}(R_i)=|G|\frac{n_i(n_i-1)}{N(N-1)}\triangleq\mu_i, \tag{1}$$

$$\mathsf{Var}_\mathsf{P}(R_i)=\mu_i(1-\mu_i)+2C\frac{n_i(n_i-1)(n_i-2)}{N(N-1)(N-2)}+(|G|(|G|-1)-2C)\frac{n_i(n_i-1)(n_i-2)(n_i-3)}{N(N-1)(N-2)(N-3)}\triangleq\sigma_i^2, \tag{2}$$

$$\mathsf{Cov}_\mathsf{P}(R_i,R_j)=(|G|(|G|-1)-2C)\frac{n_in_j(n_i-1)(n_j-1)}{N(N-1)(N-2)(N_3)}-\mu_i\mu_j, \quad \text{for } i\neq j, \tag{3}$$

where $|G|$ is the number of edges in graph G, and $C=\frac{1}{2}\sum_{k=1}^{N}|G_k|^2-|G|$ with G_k being the subgraph in G that includes all edge(s) that connect to node k, so C is the number of edge pairs that share a common node in G. Consider the following test statistic

$$S:=(R_1-\mu_1,R_2-\mu_2,\dots,R_p-\mu_p)\Sigma^{-1}\begin{pmatrix}R_1-\mu_1\\R_2-\mu_2\\\cdots\\R_p-\mu_p\end{pmatrix}, \tag{4}$$

where Σ is the covariance matrix of vector $(R_1,R_2,\dots,R_p)^T$. The following theorem, based on extension of Chen and Friedman's result, establishes the asymptotic distribution of S under multiple samples:

Theorem 1. *For an edge $e\in G$, let*

$$A_e=\{e\}\cup\{e'\in G: e' \text{ and } e \text{ share a node}\},$$

$$B_e=A_e\cup\{e''\in G:\exists\, e'\in A_e,\ \text{such that } e'' \text{ and } e \text{ share a node}\}.$$

If $|G| = O(N), \sum_{k=1}^{N} |G_k|^2 - 4|G|^2/N = O(N), |A_e||B_e| = o(N^{1.5}), \lim_{N\to\infty} n_i/N = \lambda_i \in (0,1)$*, then* $S \to \chi_p^2$ *under the permutation null.*

2.3 Simulation study: Accuracy of p-value approximation

We conducted a simulation study under $p = 4$ to evaluate the finite performance of the p-value approximation under moderate sample sizes. In particular, we compared the approximate p-value with a permutation p-value from 10,000 permutations, under different dimensions ($d = 10, 100$) and sample sizes ($N = 80, n_1 = n_2 = n_3 = n_4 = 20, N = 140, n_1 = n_2 = 20, n_3 = n_4 = 50$ and $N = 200, n_1 = n_2 = n_3 = n_4 = 50$). The data were generated from p-dimension Gaussian distribution with zero mean vector and identical covariance matrix. A k-MST ($k = 1, 3, 5$) was then constructed over the pooled observations, based on which the test statistics S could be calculated. The approximate p-value can be obtained as follows:

$$p - value = Pr(\chi^2_{df=4} > S),$$

which is to be compared with permutation p-value from 10,000 permutations.

Figure 1 and Figure 2 summarized the accuracy of the approximate p-values (approximate p-value minus permutation p-value) under different dimensions, sample sizes, and similarity graphs. It can be seen that under all conditions, the approximate p-values tend to be slightly conservative and increasing dimension leads to a slightly decreasing accuracy. In addition, using a denser similarity graph, e.g., 3-MST or 5-MST can slightly improve the p-value approximation. A sample size such that $\min_i n_i > 20$ seems to be enough in practice, in order to approximate p-value with Chi-square distribution.

3 Results

In this section ,we applied the multi-sample edge-count test to the rich TCGA data (TCGA Research Network. [2012, 2014]) and studied the roles of 186 KEGG pathways (http://www.genome.jp/kegg) in the progression of serous ovarian cancer. In TCGA, each subject is represented by multiple molecular data types including gene expression, exon expression, genotype (SNP), MicroRNA expression, copy number variation (CNV), somatic mutation, DNA methylation, as well as a complete clinical record including tumor

stage, survival, age, race, outcomes of debulking surgery and chemotherapy. To take advantage of this comprehensive data, we considered three important data types in our analysis including gene expression level, CNV and DNA methylation. Other data types could also be incorporated, without causing too much calculation.

3.1 Data preprocessing

The TCGA ovarian cancer data contains information of 17,813 genes on 565 subjects. Based on the clinical classification, four groups (stages I, II, III, IV) contain 16, 27, 438 and 84 subjects, respectively. The transcriptome profiling data, CNV data as well as the methylation data were downloaded through Genomic Data Commons (GDC) portal in January 2017. Out of 17,813 genes, 12,831 had methylation level measured for each CpG island located in their promoter regions. For genes containing more than one CpG islands, we took the average as the methylation level as suggested by TCGA Research Network. [2012]. The copy number were measured on each chromosome segment by Circular Binary Segmentation (CBS). A gene was assigned the "seg.mean" value of the segment that it falls in. If a gene spans two chromosomal segments, we took the average of the "seg.mean" value as its overall copy number. We normalized each data type for each gene to avoid possible dominance by any of these three data types.

The expression level of each gene was quantified by the count of reads mapped to the gene. The quantifications were done by software *HTSeq* of version 0.9.1 (Anders et al. [2015]) and the count data were log-transformed for further processing. In addition, we removed the effects due to different age groups and batches using a median-matching and variance-matching strategy (Hsu et al. [2012]; Zhang et al. [2014]). For example, the batch effect can be removed in the following way :

$$g^*_{ijk} = M_i + (g_{ijk} - M_{ij})\frac{\hat{\sigma}_{g_i}}{\hat{\sigma}_{g_{ij}}},$$

where g_{ijk} refers to the expression value for gene i from sample k in batch j ($j = 1,2,...,J; k = 1,2,...,n_j$), M_{ij} represents the median of $g_{ij} = (g_{ij1},...,g_{ijn})$, M_i refers to the median of $g_i = (g_{i1},...,g_{iJ})$, $\hat{\sigma}_{g_i}$ and $\hat{\sigma}_{g_{ij}}$ stand for the standard deviations of g_i and g_{ij}, respectively.

3.2 Pathway expansion and refinement

Each of the 186 pathways was expanded by integrating all the three data types. For instance, if gene *i* was in the pathway being studied, then its methylation level and CNV level were added to the pathways as two new nodes. As it is well known that the expression level of a gene could be greatly affected by genetic or epigenetic changes such as copy number variation and DNA methylation, the inclusion of these two factors may provide insights about the upstream cause of abnormal expression.

To adapt the KEGG pathways to our context, we further refined the pathways by removing genes that are irrelevant to phenotypic changes. Similar as in Edelman's et al. [2007], an F-test was used to calculate the p-value of each single gene. A gene was excluded if its p-value exceeds some predefined threshold. In this analysis, we set the threshold to be 0.1, and the same procedure was applied to the methylation and CNV data. By the refinement, the sizes of 186 KEGG pathways were greatly reduced, for instance, the ERBB pathway was reduced from 87 genes to 29 genes, and 165 out of 174 methylation/CNV nodes were excluded.

3.3 Cancer-driving pathways

For each pathway, we first conducted a multi-sample test to obtain the p-value for the whole process of development. By a Benjamini-Hochberg (BH) procedure with level 0.05, a set of nine pathways were identified, including some well-studied cancer-related pathways such as cell cycle, ERBB, p53 signaling and JAK-STAT signaling pathways. For each of the identified pathways, a two-sample test was further conducted in order to investigate the roles of these pathways in each particular transition step (I$\rightarrow$II, II$\rightarrow$III, III$\rightarrow$IV). Table 1 lists the nine cancer-driving pathways with p-values for the entire process and each particular transition. Interestingly, we found that most of these pathways contributed only to a particular step (the ERBB pathway is an exception, which has significant p-values in both transition I$\rightarrow$II and transition II$\rightarrow$III). For instance, five pathways were found to contribute only to the early-stage transition, three pathways contribute only to the high-grade transition. Six pathways, including ERBB, cell cycle, prostate cancer, TGF β signaling, pancreatic cancer and p53 signaling pathways, were found to significantly contribute to the transition I$\rightarrow$II, which confirmed some existing studies. For instance, the ERBB pathway contains important proto-oncogenes and tumor suppressors such as *PIK3C*, *KRAS* and *STAT5*. It is known that the ERBB pathway is closely related to the development of a wide variety of cancers. Especially, the excessive signaling of

growth factor receptors *ERBB1* and *ERBB2* are critical factors in the malignancy of solid tumor, and several studies have reported the critical role of ERBB in the early progression of ovarian cancer and breast cancer (TCGA Research Network. [2012]). The cell cycle pathway contains many genes that co-regulate cell proliferation, including *ATM*, *RB1*, *CCNE1* and *MYC*. Abnormal regulation among these genes may cause the over proliferation of cells and an accumulation of tumor cell numbers (TCGA Research Network. [2012]). One novel finding from our analysis is the critical roles of ECM receptor pathway in the late-stage transition (III$\rightarrow$IV). The extracellular matrix (ECM) is a major component of the local microenvironment in a cancer cell, which play important roles in cancer development (Lu et al. [2012]).

Figures 3 and 4 show the multidimensional scaling (MDS) plots (with Manhattan distance) using stage I and stage II samples, based on the refined gene sets of ERBB pathway (p-value$= 7.2 \times 10^{-5}$) and cell cycle pathway (p-value$= 4.0 \times 10^{-6}$), respectively. It can be seen that both pathways well differentiate the two groups, indicating their substantial involvement in this particular step of transition. Figures 5 and 6 display the MDS plots for stage III and stage IV samples, based on the refined gene sets of ECM receptor pathway (p-value$= 6.2 \times 10^{-5}$) and apoptosis pathway (p-value$= 1.9 \times 10^{-3}$), respectively. As compared to III$\rightarrow$IV transition, the transition from stage I to stage II is more substantial, which partially confirm some existing studies and clinical statistics. For instance, the 5-year survival rate for stage-I ovarian cancer patients is above 90%, while the rate for stage-II cancer patients drop below 30% (TCGA Research Network. [2011]), suggesting an intrinsic genetic/epigenetic difference between these two groups. As a contrast, we also presented two MDS plots with insignificant p-valules in Figure 7 (p-value=0.3, prostate cancer pathway) and Figure 8 (p-value=0.6, pancreatic cancer pathway).

The regulatory relations of the genes in the refined set were statistically predicted by a logistic Bayesian network model (Zhang et al. [2014]). A coordinate descent algorithm was employed to solve the penalized likelihood function and the penalizing constant λ was tuned by a likelihood method suggested by Zhang et al. (2014). Figures 9 and 10 show the predicted Bayesian networks for the cell cycle pathway and ERBB pathway. These directed networks may provide more clues about how the cancer-driving genes regulate each other, and together change the phenotype. For instance, in the cell cycle pathway (Figure 9), we found gene *CDC20* is a driver gene (high outdegree and high centrality), regulating eight other cancer-driving genes in the network. Intervention on this gene may result in a global change of this network.

4 Discussion

Gene set analyses hold great promise in elucidating the molecular basis of complex diseases such as cancers. Nevertheless, most of the existing gene set analyses are knowledge-based and relied on the enrichment analysis of the pathways defined in existing databases. In this paper, we proposed a data-driven tool for gene set analysis, without requiring any predefined pathways or gene sets. The new test is very easy to implement in practice as it only requires the calculation of a similarity graph over the observations. In practice, one can apply our test to large data set as an efficient searching tool for phenotype-changing pathways, as the computation is simple and efficient. Moreover, under the permutation null and some mild conditions, the test statistics converges to a Chi-square distribution and sample size in tens for each group is generally good enough for p-value approximation, as we show in the simulation study.

The new test also has some limitations. First, the new test only targets for the difference of multiple joint distributions, but cannot detect the direction of changes. A follow-up test or a graphical visualization will be needed in order to tell the direction of changes, i.e., the up-regulation or down-regulation. For example, one could calculate an enrichment score for the pathway being studied as in existing gene set analyses or use a heatmap of the gene set.

Second, as we illustrated in the TCGA application, the performance of the new method relies on a refinement of the gene set, as the existence of irrelevant genes may greatly affect the identification of genes that differentiate phenotypes. In a case that the majority of a gene set are uncorrelated with phenotypic change, the test will fail to identify that gene set. One possible way to solve this problem is redefining distance metric, for example, one can consider to use some weighted distance measures or the ℓ_∞-norm measures that are more sensitive to a change in single gene or a small proportion of genes.

5 Conclusions

In this work, we introduced a simple but powerful multivariate test to identify (epi)genetic pathways that drive the cancer progression. Different from most existing gene set analyses, our test is data-driven and nonparametric, with allowance of user-defined gene sets or pathways. As we see in the method section, this graph-based test can be easily implemented by calculating Euclidian distance between observations and constructing a minimum spanning tree, thus it is efficient to high dimensional problems. Besides the cancer

progression application presented in this paper, the new test can be also applied to many other problems. For instance, one can test for pathways that differentiate cancer subtypes.

Acknowledgement

QZ's research is supported in part by the Arkansas Biosciences Institute, the major research component of the Arkansas Tobacco Settlement Proceeds Act of 2000. HC's research is supported by ???.

Competing Interests

The authors have declared that no competing interests exist.

Abbreviations

TCGA - The Cancer Genome Atlas; MST - Minimum spanning tree; CNV - Copy number variation; BH - Benjamini-Hochberg; MDS - Multidimensional scaling; BN - Bayesian network

References

Al-Shahrour, F., Arbiza, L., Dopazo, H., Huerta-Cepas, J., Mingues, P. et al. (2007), From genes to functional classes in the study of biological systems, *BMC Bioinformatics*, **8**(114)

Anders, S., Pyl, P.T. & Huber, W. (2015), HTSeq - a Python framework to work with high-throughput sequencing data, *Bioinformatics*, **31**, 166-169.

Backes, C., Keller, A., Kuentzer, J., Kneissl, B., Comtesse, N. et al. (2007), GeneTrail-advanced gene set enrichment analysis, *Nucleic Acid Research*, **35**, W186-W192

Breslin, T., Edén, P. & Krogh, M. (2004), Comparing functional annotation analyses with Catmap, *BMC Bioinformatics*, **5**(193)

Cai, T., Liu, W. & Xia, Y. (2014), Two-sample test of high dimensional means under dependence, *Journal of the Royal Statistical Society - Series B*, **76**(2), 349-72.

Chen, H.Y. & Shao, Q. (2005), Stein's method for normal approximation, *Lecture Note Series, IMS, NUS*

Chen, H. & Friedman, J.H. (2017), A new graph-based two-sample test for multivariate and object data, *Journal of the American Statistical Association*, **112**, 397-409.

Cheriton, D. & Tarjan, R. (2006). Finding minimum spanning trees, *SIAM Journal of Computing*, **5**(4), 724-742.

Edelman, E., Guinney, J., Chi, J.-T., Febbo, P.G., Mukherjee, S. (2008), Modeling cancer progression via pathway dependencies, *PLoS Computational Biology*, **4**(2), e28.

Friedman, J.H. & Rafsky, L.C. (1979), Multivariate generalizations of the Wald-Wolfowitz and Smirnov two-sample tests, *Annals of Statistics*, **7**(4), 697-717

Glaab, E., Baudot, A., Krasnogor, N., Schneider, R., Valencia, A. (2012), EnrichNet: network-based gene set enrichment analysis, *Bioinformatics*, **28**(18), i451-i457

Gregory, K., Carroll, R.J., Baladandayuthapani, V. & Lahiri, S.N. (2015), A two-sample test for equality of means in high dimension, *Journal of the American Statistical Association*, **110**(510), 837-49.

Hoek, K.S., Schlegel, N.C., Brafford, P., Sucker, A., Ugurel, S. et al. (2006), Metastatic potential of melanomas defined by specific gene expression profiles with no BRAF signature, *Pigment Cell Research*, **19**(4), 290 - 302.

Hsu, F., Serpedin, E., Hsiao, T., Bishop, A., Dougherty, E., et al. (2012), Reducing confounding and suppression effects in tcga data: an integrated analysis of chemotherapy response in ovarian cancer, *BMC Genomics*, **13**(S13)

Ivshina, A.V., George, J., Senko, O., Mow, B., Putti, T.C. et al. (2006), Genetic reclassification of histologic grade delineates new clinical subtypes of breast cancer, *Cancer Research*, **66**(21), 10292 -301.

Kim, S. & Volsky, D.J. (2005), PAGE: Parametric analysis of gene set enrichment, *BMC Bioinformatics*, **6**(144)

Lee, H.K., Braynen, W., Kehsav, K., Pavlidis, P. (2005), ErmineJ: Tool for functional analysis of gene expression data sets, *BMC Bioinformatics*, **6**(269)

Lopes, R.H.C., Hobson, P.R. & Reid, I.D. (2008), Computationally efficient algorithms for the two-dimensional Kolmogorov-Smirnov test, *Journal of Physics: Conference Series*, **19**(4)

Lu, P., Weaver, V.M. & Werb, Z. (2012), The extracellular matrix: A dynamic niche in cancer progression, *Journal of Cell Biology*, **196**(4):395.

Luo, W., Friedman, M.S., Shedden, K., Hankenson, K.D., Woolf, P.J. (2009), GAGE: generally applicable gene set enrichment for pathway analysis, *BMC Bioinformatics*, **10**(161)

Ma, X., Salunga, R., Tuggle, J.T., Gaudet, J., Enright, E. et al. (2003), Gene expression profiles of human breast cancer progression, *Proceedings of the National Academy of Sciences of USA*, **100**(10), 5974 - 9.

Pancione, M., Remo, A. & Colantuoni, V. (2012), Genetic and epigenetic events generateMultiple pathways in colorectal cancer progression, *Pathology Research International*, **2012**, 509348.

Rosenbaum, P.R. (2005), An exact distribution-free test comparing two multivariate distributions based on adjacency, *Journal of Royal Statistical Society, Series B*, **67**(4), 515-530

Smith, A.P., Hoek, K. & Becker, D. (2005), Whole-genome expression profiling of the melanoma progression pathway reveals marked molecular differences between nevi/melanoma in situ and advanced-stage melanomas, *Cancer Biology and Therapy*, **4**(9), 1018 - 29.

Srivastava, M.S., Katayama, S. & Kano, Y. (2013), A two sample test in high dimensional data, *Journal of Multivariate Analysis*, **114**, 349-58.

Subramanian, A., Tamayo, P., Mootha, V., Mukherjee, S., Ebert, B.L. et al. (2005), Gene set enrichment analysis: A knowledge-based approach for interpreting genome-wide expression profiles, *Proceedings of the National Academy of Sciences of USA*, **102**(43), 15545-50.

Talantov, D., Mazumder, A. Yu, J., Briggs, T., Jiang, Y. et al. (2005), Novel genes associated with malignant melanoma but not benign melanocytic lesions, *Clinical Cancer Research*, **11**(20), 7234 - 42.

The Cancer Genome Atlas Research Network. (2008), Comprehensive molecular portraits of human breast tumours, *Nature*, **490**, 61-70.

The Cancer Genome Atlas Research Network. (2014), Comprehensive molecular profiling of lung adenocarcinoma, *Nature*, **511**, 543-550.

The Cancer Genome Atlas Research Network. (2011), Integrated genomic analyses of ovarian carcinoma, *Nature*, **474**, 609-15.

Tomlins, S.A., Mehra, R., Rhodes, D.R., Cao, X., Wang, L. et al. (2007), Integrative molecular concept modeling of prostate cancer progression, *Nature Genetics*, **39**(1), 41 - 51.

Tu, K., Yu, H. & Zhu, M. (2005), MEGO: gene functional module expression based on gene ontology, *BioTechniques*, **38**, 277-283

Zhang, Q., Burdette, J.E. & Wang, J.-P. (2014), Integrative network analysis of tcga data for ovarian cancer, *BMC Systems Biology*, **8**(1338), 1-18.

Appendix: Detailed proof for asymptotic distribution of the test statistics

Proof. To prove the theorem, we take one step back to study the statistic under the bootstrap null distribution, which is defined as follows: For each observation, we assign it to be from sample i with probability n_i/N independently of other observations. Let X_i be the number of observations that are assigned to be from sample i. Then, conditioning on $\{X_i = n_i\}_{i=1,\dots,p}$, the bootstrap null distribution becomes the permutation null distribution. We use $\mathsf{P_B}$, $\mathsf{E_B}$, $\mathsf{Var_B}$ to denote the probability, expectation, and variance under the bootstrap null distribution, respectively.

We have

$$\mathsf{E_B}(R_i) = \frac{n_i^2}{N^2}|G| \triangleq \mu_i^{\mathsf{B}},$$
$$\mathsf{Var_B}(R_i) = \frac{n_i^2(N-n_i)^2}{N^4}|G| + \frac{n_i^3(N-n_i)}{N^4}\sum_{k=1}^{N}|G_k|^2 \triangleq (\sigma_i^{\mathsf{B}})^2.$$

Let

$$W_i^{\mathsf{B}} = \frac{R_i - \mu_i^{\mathsf{B}}}{\sigma_i^{\mathsf{B}}}, \quad W_i = \frac{R_i - \mu_i}{\sigma_i},$$
$$U_i = \frac{X_i - n_i}{\sqrt{N\lambda_{i,N}(1-\lambda_{i,N})}},$$

where $\lambda_{i,N} = n_i/N$. Under the conditions in the theorem, as $N \to \infty$, we can prove the following results:

(1) Under the bootstrap null, $(W_1^{\mathsf{B}}, W_2^{\mathsf{B}}, \dots, W_p^{\mathsf{B}}, U_1, \dots, U_{p-1})$ becomes multivariate normal distributed and the covariance matrix of $(U_1, \dots, U_{p-1})$ is of positive definite.

(2)
$$\frac{\sigma_i^{\mathsf{B}}}{\sigma_i} \to c_i, \quad \frac{\mu_i^{\mathsf{B}} - \mu_i}{\sigma_i^{\mathsf{B}}} \to 0,$$

where c_i's are constants.

(3) $rank(\Sigma) = p$.

Given (1), the conditional distribution of $(W_1^{\mathsf{B}}, W_2^{\mathsf{B}}, \dots, W_p^{\mathsf{B}})'$ given $(U_1, \dots, U_{p-1})$ becomes a multivariate Gaussian distribution under the boostrap null distribution as $N \to \infty$. Since the permutation null distribution

is equivalent to the bootstrap null distribution given $U_i = 0, i = 1, \ldots, p-1$, $(W_1^{\mathsf{B}}, W_2^{\mathsf{B}}, \ldots, W_p^{\mathsf{B}})'$ becomes a multivariate Gaussian distribution under the permutation null distribution as $N \to \infty$. Since

$$W_i = \frac{\sigma_i^{\mathsf{B}}}{\sigma_i}\left(W_i^{\mathsf{B}} + \frac{\mu_i^{\mathsf{B}} - \mu_i}{\sigma_i^{\mathsf{B}}}\right),$$

given (2), we have $(W_1, W_2, \ldots, W_p)'$ becomes a multivariate Gaussian distribution under the permutation null distribution as $N \to \infty$. Together with (3), we have the conclusion in the theorem.

In the following, we prove the results (1), (2) and (3).

To prove the first part of (1), by by Cramér-Wold device, we only need to show that $W = \sum_{i=1}^{p} a_i W_i^{\mathsf{B}} + \sum_{i=1}^{p-1} b_i U_i$ is asymptotically Gaussian distribution for any combination of a_i's and b_i's such that $\mathsf{Var}_{\mathsf{B}}(W) > 0$.

To show this, we use Stein's method. Consider sums of the form $V = \sum_{i \in \mathcal{J}} \xi_i$, where $\mathcal{J}$ is an index set and ξ are random variables with $\mathsf{E}\xi_i = 0$, and $\mathsf{E}(V^2) = 1$. The following assumption restricts the dependence between $\{\xi_i :\ i \in \mathcal{J}\}$.

Assumption 1. *[Chen and Shao , 2005, p. 17] For each $i \in \mathcal{J}$ there exists $K_i \subset L_i \subset \mathcal{J}$ such that ξ_i is independent of $\xi_{K_i^c}$ and ξ_{K_i} is independent of $\xi_{L_i^c}$.*

We will use the following theorem in proving Theorem 1.

Theorem 2. *[Chen and Shao , 2005, Theorem 3.4] Under Assumption 1, we have*

$$\sup_{h \in Lip(1)} |Eh(V) - Eh(Z)| \leq \delta,$$

where $Lip(1) = \{h : \mathbb{R} \to \mathbb{R}; \|h'\| \leq 1\}$, Z has $\mathcal{N}(0,1)$ distribution and

$$\delta = 2\sum_{i \in \mathcal{J}} (E|\xi_i \eta_i \theta_i| + |E(\xi_i \eta_i)|E|\theta_i|) + \sum_{i \in \mathcal{J}} E|\xi_i \eta_i^2|$$

with $\eta_i = \sum_{j \in K_i} \xi_j$ and $\theta_i = \sum_{j \in L_i} \xi_j$, where K_i and L_i are defined in Assumption 1.

For $e \in G$, let

$$\xi_e = \sum_{i=1}^{p} a_i \frac{I_{J_e = i} - \lambda_{i,N}}{\sigma_i^{\mathsf{B}}},$$

where $\{J_e = i\}$ means the edge connects two observations from sample i.

For $k \in \{1, \dots, N\}$, let

$$\xi_k = \sum_{i=1}^{p-1} b_i \frac{I_{g_k=i} - \lambda_{i,N}}{\sqrt{N\lambda_{i,N}(1-\lambda_{i,N})}},$$

where $\{g_k = i\}$ means node k is from sample i.

Then $W = \sum_{e\in G} \xi_e + \sum_{k=1}^{N} \xi_k$. We have $\mathsf{E}_\mathsf{B}(\xi_e) = 0, \forall e \in G$ and $\mathsf{E}_\mathsf{B}(\xi_k) = 0, \forall k \in \{1, \dots, N\}$.

Let $a = \max(\max_{i\in\{1,\dots,p\}} |a_i|, \max_{i\in\{1,\dots,p-1\}} |b_i|)$, $\sigma = \min(\min_{i\in\{1,\dots,p\}} \sigma_i^\mathsf{B}, \min_{i\in\{1,\dots,p-1\}} \sqrt{N\lambda_{i,N}(1-\lambda_{i,N})}) = O(\sqrt{N})$. Then $|\xi_e|, |\xi_k| \leq pa/\sigma, \forall e \in G, i \in \{1, \dots, N\}$. Also, since $|G| = O(N)$, we have $\sigma = O(\sqrt{N})$.

For $e = (e_-, e_+) \in G$, let

$$K_e = A_e \cup \{e_-, e_+\},$$
$$L_e = B_e \cup \{\text{nodes in } A_e\}.$$

Then K_e and L_e satisfy Assumption 1.

For $k \in \{1, \dots, N\}$, let

$$K_k = \{e \in G_k\} \cup \{i\},$$
$$L_k = \{e \in G_{k,2}\} \cup \{\text{nodes in } G_i\}.$$

Then K_k and L_k satisfy Assumption 1.

Let $\mathcal{J} = \{e : e \in G\} \cup \{1, \dots, N\}$.

For $j \in \mathcal{J}$, let $\eta_j = \sum_{k\in K_j} \xi_k, \theta_j = \sum_{k\in L_j} \xi_k$. By Theorem 2, we have $\sup_{h\in Lip(1)} |\mathsf{E}h(W) - \mathsf{E}h(Z)| \leq \delta$ for

$Z \sim \mathcal{N}(0,1)$, where

$$\begin{aligned}
\delta &= \frac{1}{\sqrt{\mathsf{Var}_{\mathsf{B}}(W)}}\left(2\sum_{j\in\mathcal{J}}(\mathsf{E}_{\mathsf{B}}|\xi_j\eta_j\theta_j| + |\mathsf{E}_{\mathsf{B}}(\xi_j\eta_j)|\mathsf{E}_{\mathsf{B}}|\theta_j|) + \sum_{j\in\mathcal{J}}\mathsf{E}_{\mathsf{B}}|\xi_j\eta_j^2|\right)\\
&\le \frac{1}{\sqrt{\mathsf{Var}_{\mathsf{B}}(W)}}\left(5\sum_{e\in G}\frac{p^3a^3}{\sigma^3}(|A_e|+2)(|B_e|+|A_e|+1) + 5\sum_{k=1}^{N}(|G_i|+1)(|G_{i,2}|+1)\right)\\
&\le \frac{1}{\sqrt{\mathsf{Var}_{\mathsf{B}}(W)}}\frac{90p^3a^3}{\sigma^3}\sum_{e\in G}|A_e||B_e|
\end{aligned}$$

Since $\sigma = O(\sqrt{N})$, when $|A_e||B_e| = o(N^{1.5})$, we have $\delta \to 0$ as $N \to \infty$.

We next check the covariance matrix of $(U_1,\ldots,U_{p-1})$. The diagonal elements of this matrix are all 1's and the off-diagonal element $(i,j), i \neq j$ is

$$-\sqrt{\frac{\lambda_{i,N}\lambda_{j,N}}{(1-\lambda_{i,N})(1-\lambda_{j,N})}}.$$

So the covariance matrix can be written as $D - vv^T$ where D is a diagonal matrix with the ith diagonal element $1+\lambda_{i,N}/(1-\lambda_{i,N})$, and $v = (\sqrt{\lambda_{1,N}/(1-\lambda_{1,N})},\ldots,\sqrt{\lambda_{p-1,N}/(1-\lambda_{p-1,N})})^T$. Since $1 - v^TD^{-1}v = 1-\sum_{i=1}^{p-1}\lambda_{i,N} = \lambda_{p,N} \neq 0$, $D - vv^T$ is invertible. Since the covariance matrix is by natural non-negative definite, when it is full rank, it is positive definite.

We next show result (2). Notice that σ_i^2 can be re-written as

$$\sigma_i^2 = \frac{n_i(n_i-1)(N-n_i)(N-n_i-1)}{N(N-1)(N-2)(N-3)}\left(|G| + \frac{n_i-2}{N-n_i-1}\left(\sum_{k=1}^{N}|G_i|^2 - \frac{4|G|^2}{N}\right) - \frac{2}{N(N-1)}|G|^2\right).$$

Since $|G|, \sum_{k=1}^{N}|G_k|^2 - 4|G|^2/N = O(N)$, let $\lim_{N\to\infty}|G|/N = a_0$ and $\lim_{N\to\infty}(\sum_{k=1}^{N}|G_k|^2 - 4|G|^2/N)/N = b_0$, then

$$\lim_{N\to\infty}\sigma_i^2/N = \lambda_i^2(1-\lambda_i)^2(a_0 + b_0\lambda_i/(1-\lambda_i)),$$

$$\lim_{N\to\infty}(\sigma_i^{\mathsf{B}})^2/N = \lambda_i^2(1-\lambda_i)^2a_0 + \lambda_i^3(1-\lambda_i)(b_0+4a_0^2).$$

Hence

$$\lim_{N\to\infty} \frac{\sigma_i^{\mathsf{B}}}{\sigma_i} = \sqrt{1 + \frac{4a_0^2\lambda_i}{a_0(1-\lambda_i)+b_0\lambda_i}}.$$

Also, since $\mu_i^{\mathsf{B}} - \mu_i = |G|\frac{n_i(N-n_i)}{N^2(N-1)}$, so

$$\lim_{N\to\infty} \frac{\mu_i^{\mathsf{B}} - \mu_i}{\sigma_i^{\mathsf{B}}} = \lim_{N\to\infty} \frac{a_0\lambda_i(1-\lambda_i)}{\sigma_i^{\mathsf{B}}} = 0.$$

We next show (3). The diagonal elements of Σ are σ_i^2's. The off-diagonal elements are, for $i \neq j$,

$$\Sigma[i,j] = \frac{n_i n_j (n_i - 1)(n_j - 1)}{N(N-1)(N-2)(N-3)} \left(|G| - \left(\sum_{k=1}^{N} |G_k|^2 - \frac{4|G|^2}{N} \right) - \frac{2}{N(N-1)}|G|^2 \right).$$

Then, the leading terms of Σ can be decomposed as $\tilde{D} + (a_0 - b_0)uu^T$, where $\tilde{D}$ is a diagonal matrix with $\tilde{D}[i,i] = \lambda_i^2((1-2\lambda_i)a_0 + \lambda_i b_0)$, and $u = (\lambda_1^2, \ldots, \lambda_p^2)^T$. Since $1 + (a_0 - b_0)u^T\tilde{D}^{-1}u = 1 + \sum_{i=1}^{p} \frac{\lambda_i^2(a_0-b_0)}{(1-2\lambda_i)a_0+\lambda_i b_0}$ is strictly larger than 0 (this quantity is strictly increasing in a_0 and equals 0 when $a_0 = 0$), Σ is of full rank.

□

Tables and Figures

Table 1: Pathways that drive ovarian cancer progression

	Pathway	p (overall)	p (I→II)	p (II→III)	p (III→IV)
1	ERBB	4.4×10^{-7}	$\mathbf{7.2\times10^{-5}}$	$\mathbf{3.8\times10^{-2}}$	2.0×10^{-1}
2	Cell cycle	9.6×10^{-7}	$\mathbf{4.0\times10^{-6}}$	5.3×10^{-1}	9.4×10^{-2}
3	Prostate cancer	4.1×10^{-6}	$\mathbf{2.5\times10^{-4}}$	1.1×10^{-1}	3.0×10^{-1}
4	ECM receptor	1.0×10^{-5}	1.7×10^{-1}	7.7×10^{-1}	$\mathbf{6.2\times10^{-5}}$
5	TGF β signaling	3.9×10^{-4}	$\mathbf{2.7\times10^{-3}}$	6.1×10^{-1}	7.9×10^{-1}
6	Apoptosis	6.2×10^{-4}	1.8×10^{-1}	8.5×10^{-1}	$\mathbf{1.9\times10^{-3}}$
7	Pancreatic cancer	6.9×10^{-4}	$\mathbf{3.3\times10^{-3}}$	7.2×10^{-2}	6.0×10^{-1}
8	P53 signaling	7.5×10^{-4}	$\mathbf{9.1\times10^{-4}}$	4.0×10^{-1}	5.5×10^{-4}
9	JAK-STAT signaling	1.4×10^{-3}	4.9×10^{-1}	8.2×10^{-1}	$\mathbf{1.6\times10^{-2}}$

Presented in the table are the list of pathways identified by the new test. The columns represent pathway name, overall p-value and p-value for each particular transition step, i.e., I→II, II→III, III→IV.

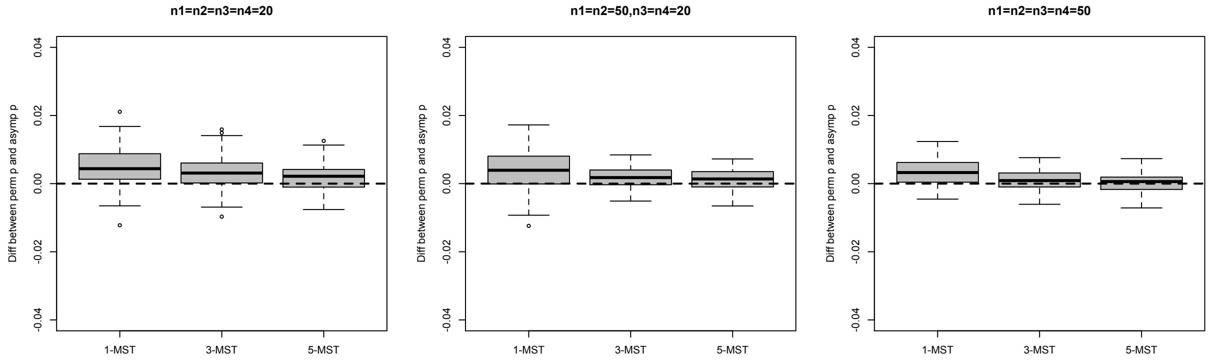

Figure 1: Boxplots of the difference between approximate p-value and permutation p-value (approximate p minus permutation p), under different sample sizes and similarity graph construction (1-MST, 3-MST and 5-MST). Dimension d is 10.

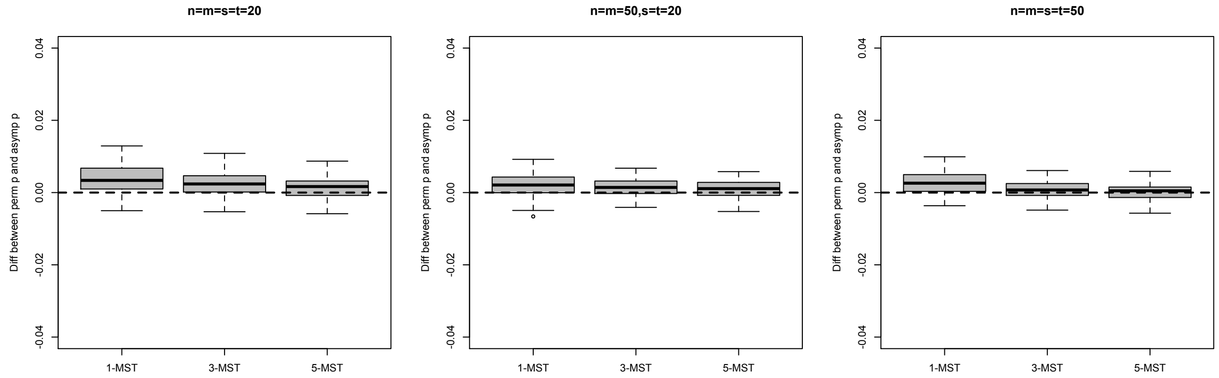

Figure 2: Boxplots of the difference between approximate p-value and permutation p-value (approximate p minus permutation p), under different sample sizes and similarity graph construction (1-MST, 3-MST and 5-MST). Dimension d is 100.

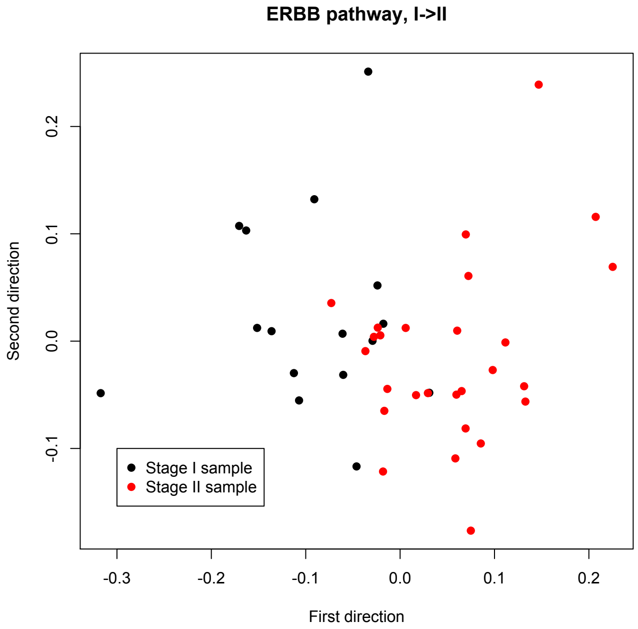

Figure 3: Multidimensional scaling (MDS) plot for subjects at stage I and stage II, based on the refined gene set of ERBB pathway.

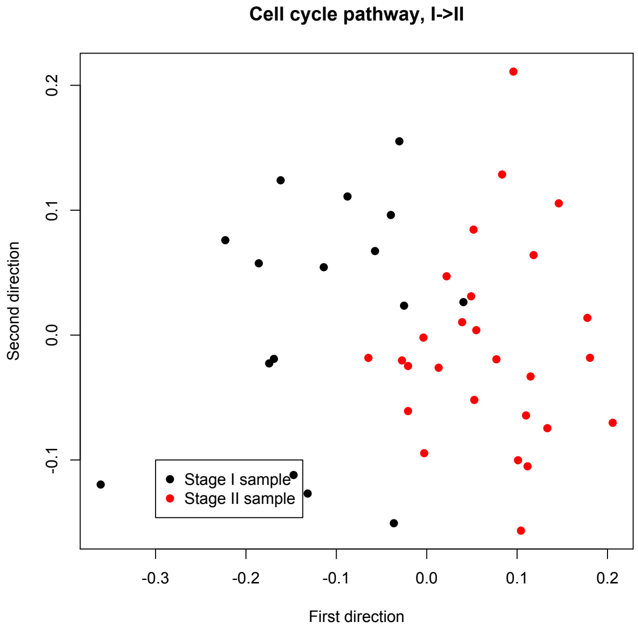

Figure 4: Multidimensional scaling (MDS) plot for subjects at stage I and stage II, based on the refined gene set of cell cycle pathway.

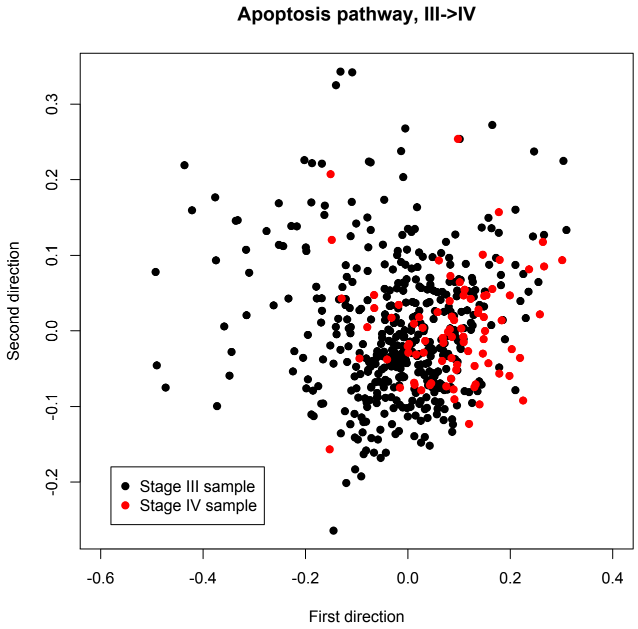

Figure 5: Multidimensional scaling (MDS) plot for subjects at stage III and stage IV, based on the refined gene set of apoptosis pathway.

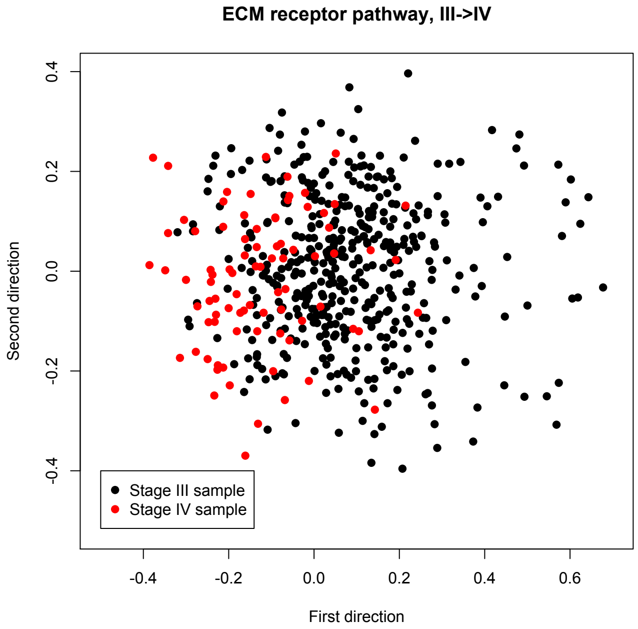

Figure 6: Multidimensional scaling (MDS) plot for subjects at stage III and stage IV, based on the refined gene set of ECM receptor pathway.

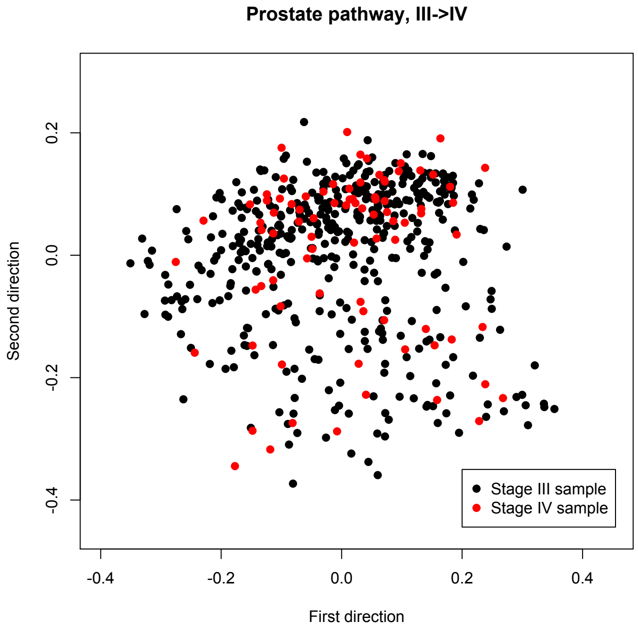

Figure 7: Multidimensional scaling (MDS) plot for subjects at stage III and stage IV, based on the refined gene set of prostate cancer pathway.

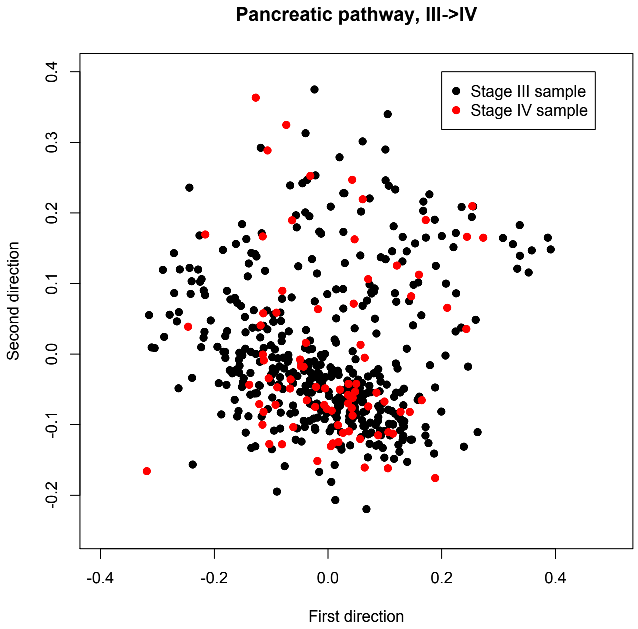

Figure 8: Multidimensional scaling (MDS) plot for subjects at stage III and stage IV, based on the refined gene set of pancreatic cancer pathway.

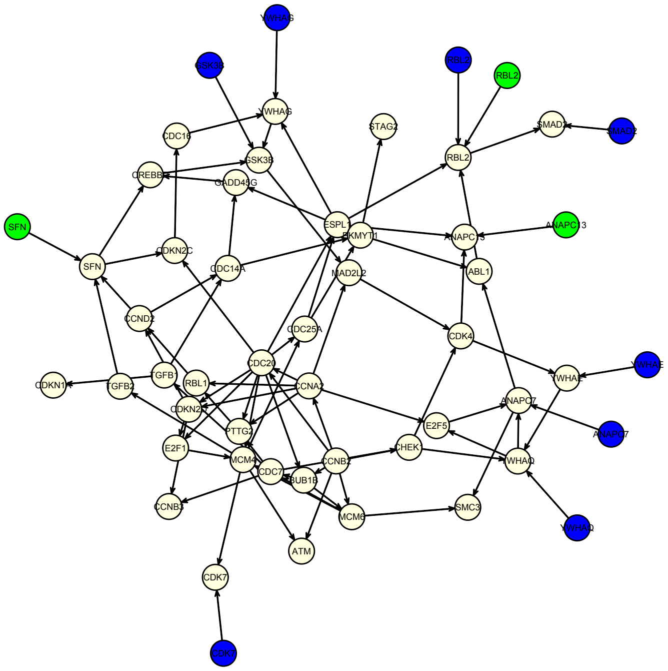

Figure 9: Causal network for the refined cell cycle pathway, predicted by the logistic Bayesian network model introduced in Zhang, Burdette and Wang (2014), where each yellow node represents the expression level of a gene, each green node represents the overall methylation level of a gene, and each blue node represents the copy number of a gene.

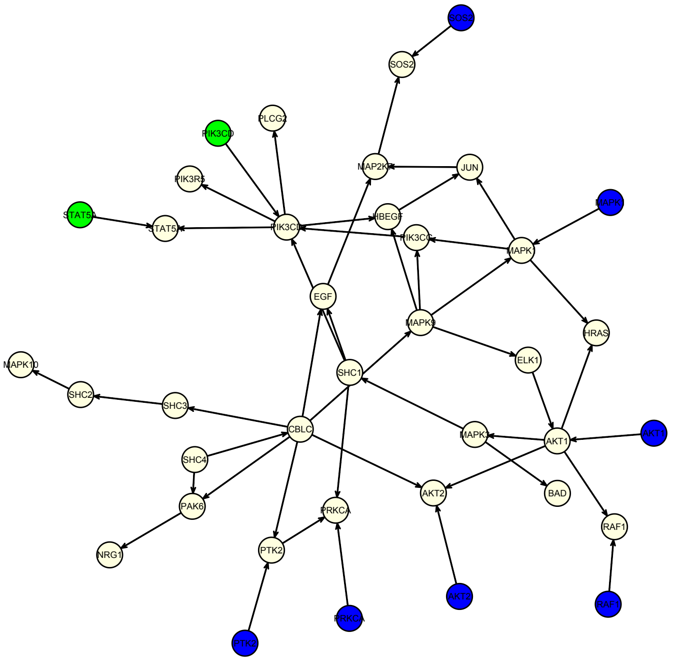

Figure 10: Causal network for the refined ERBB pathway, predicted by the logistic Bayesian network model introduced in Zhang, Burdette and Wang (2014), where each yellow node represents the expression level of a gene, each green node represents the overall methylation level of a gene, and each blue node represents the copy number of a gene.
